# Growth hormone induces mitotic catastrophe of glomerular podocytes and contributes to proteinuria

**DOI:** 10.1038/s41419-021-03643-6

**Published:** 2021-04-01

**Authors:** Rajkishor Nishad, Dhanunjay Mukhi, Ashish Kumar Singh, Manga Motrapu, Kumaraswami Chintala, Prasad Tammineni, Anil K. Pasupulati

**Affiliations:** 1 Department of Biochemistry, School of Life Sciences, University of Hyderabad, Hyderabad, 500046 India; 2 Department of Animal Biology, School of Life Sciences, University of Hyderabad, Hyderabad, 500046 India

**Keywords:** Podocytes, Diabetic nephropathy

## Abstract

Glomerular podocytes are integral members of the glomerular filtration barrier in the kidney and are crucial for glomerular permselectivity. These highly differentiated cells are vulnerable to an array of noxious stimuli that prevail in several glomerular diseases. Elevated circulating growth hormone (GH) levels are associated with podocyte injury and proteinuria in diabetes. However, the precise mechanism(s) by which excess GH elicits podocytopathy remains to be elucidated. Previous studies have shown that podocytes express GH receptor (GHR) and induce Notch signaling when exposed to GH. In the present study, we demonstrated that GH induces TGF-β1 signaling and provokes cell cycle reentry of otherwise quiescent podocytes. Though differentiated podocytes reenter the cell cycle in response to GH and TGF-β1, they cannot accomplish cytokinesis, despite karyokinesis. Owing to this aberrant cell cycle event, GH- or TGF-β1-treated cells remain binucleated and undergo mitotic catastrophe. Importantly, inhibition of JAK2, TGFBR1 (TGF-β receptor 1), or Notch prevented cell cycle reentry of podocytes and protected them from mitotic catastrophe associated with cell death. Inhibition of Notch activation prevents GH-dependent podocyte injury and proteinuria. Similarly, attenuation of GHR expression abated Notch activation in podocytes. Kidney biopsy sections from patients with diabetic nephropathy (DN) show activation of Notch signaling and binucleated podocytes. These data indicate that excess GH induced TGF-β1-dependent Notch1 signaling contributes to the mitotic catastrophe of podocytes. This study highlights the role of aberrant GH signaling in podocytopathy and the potential application of TGF-β1 or Notch inhibitors, as a therapeutic agent for DN.

## Introduction

Glomerular complications are the predominant cause of end-stage kidney disease, and clinical conditions, such as diabetes and hypertension are associated with glomerular dysfunction and proteinuria. Glomerular podocytes are highly differentiated specialized visceral cells that account for ~30% of glomerular cells. These cells provide epithelial coverage to the capillaries and together with glomerular basement membrane (GBM) and perforated endothelial cells, constitute a glomerular filtration barrier. The unique cytoplasmic extensions of podocytes are known as foot processes, which attach to the GBM and interdigitate with neighboring foot processes to form the slit diaphragm (SD). The sophisticated architecture of SD contributes to the glomerular permselectivity. The process of progressive podocyte damage characterized by podocyte hypertrophy, detachment of podocytes, and, finally, irreversible loss of podocytes has been observed in human and experimental models of nephropathy and glomerular diseases^1^. Injury and depletion of podocytes, leading to podocyte insufficiency and capillary collapse, have been implicated in glomerulosclerosis and resulting chronic kidney disease. Since matured podocytes are terminally differentiated and quiescent^2^, injured podocytes leave denuded areas on the glomerular capillary, which results in albuminuria^3,4^.

Albuminuria is a marker for renal dysfunction in the general population, and is an early marker for overt nephropathy in patients with diabetes mellitus. Elevated circulating growth hormone (GH) levels and increased renal expression of the GH receptor (GHR) are associated with nephropathy in poorly controlled type 1 diabetes^5,6^. Other excess GH conditions, such as acromegaly, are characterized by glomerular hypertrophy, sclerosis, and albuminuria. Conversely, blunting GH action is protective for glomerulopathy^7^. In an earlier report, we demonstrated that podocytes express GHR and respond to exogenous GH via activation of canonical JAK–STAT signaling^8^. Our previous work showed that GH activates Notch signaling^9^ and promotes epithelial to mesenchymal transition (EMT) of podocytes by inducing ZEB2 (zinc finger E-box binding homeobox 2), also known as smad-interacting protein1^10,11^.

Interestingly, GH-induced glomerulosclerosis and interstitial fibrosis in diabetic rats are associated with increased TGF-β1 levels^12^, whereas inhibition of JAK2, an immediate downstream target of GH, reduced TGF-β1 expression^13^. Although multiple studies revealed TGF-β1’s role in morphologic manifestations and clinical characteristics of diabetic nephropathy (DN), the stimuli that activate the TGF-β/SMAD pathway in the podocyte remain unclear^13,14^. In the present study, we demonstrate that GH induces TGF-β1 expression, resulting in Notch activation. GH and TGF-β1-dependent Notch activation stimulated podocyte reentry into the cell cycle. However, the persistent activation of Notch signaling resulted in unproductive cytokinesis and mitotic catastrophe-induced cell death.

## Results

### GH induces TGF-β1 and cognate TGF-β–SMAD pathway in podocytes

**Fig. 1:**
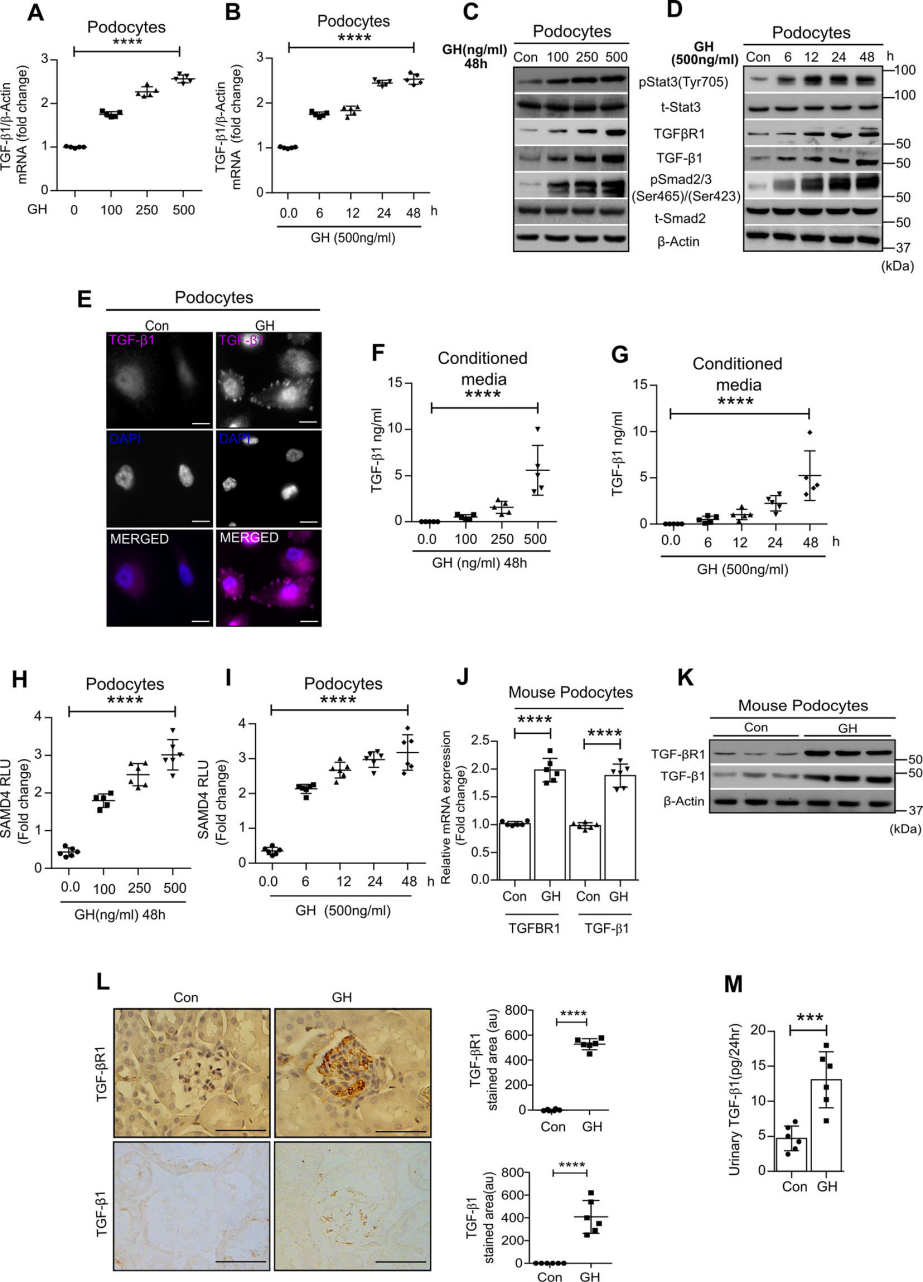
GH induces TGF-β/SMAD pathway in podocytes. **A**, **B** qRT-PCR analysis showing the expression of TGF-β1 in human podocytes treated with or without GH in concentration (100–500 ng/ml) and time- (up to 48 h) dependent manner. mRNA levels were normalized to β-actin and presented as fold change on the *y*-axis. Mean ± SD. (*n* = 5). ****p < 0.0001 by one-way ANOVA post hoc Dunnett test. **C**, **D** Immunoblotting analysis for indicated genes from podocytes treated with GH in concentration (100–500 ng/ml) and time- (up to 48 h) dependent manner. (*n* = 3). **E** Immunofluorescence analysis for TGF-β1 (purple color) and counterstained with DAPI [4′,6-diamidino-2-phenylindole (blue color)] in podocytes treated with or without GH (500 ng/ml, 48 h). Scale bar = 50 μm, magnification ×500. **F**, **G** Estimation of TGF-β1 in conditioned media (CM) from podocytes treated with GH in concentration (100–500 ng/ml) and time- (up to 48 h) dependent manner. Mean ± SD. (*n* = 5). **H**, **I** SMAD4 luciferase activity in podocytes treated with GH in concentration (100–500 ng/ml) and time- (up to 48 h) dependent manner. Mean ± SD. (*n* = 6). **F**–**I** ****p < 0.0001 by one-way ANOVA post hoc Dunnet test. **J**, **K** qRT-PCR and immunoblotting analysis show TGFBR1 and TGF-β1 in mice podocytes treated with or without GH (1.5 mg/kg b.w). Mean ± SD. (*n* = 3). ****p < 0.0001 by Student’s *t* test. Each data point represents the average value of mice from each group. *n* = 6. **L** Representative images for TGFBR1 and TGF-β1 expression assessed by 3,3′-diaminobenzidine (DAB) staining in mice glomerular sections treated with GH. Scale bar = 100 μm, magnification ×200. (*n* = 3). Quantification of TGF-βR1 and TGF-β1 staining area in the glomerulus (right panel) represented as a dot plot. Mean ± SD. (*n* = 6). ****p < 0.0001 by Student’s *t* test. **M** Quantification of TGF-β1 in urine from mice administered with GH. Mean ± SD. (*n* = 6). ****p < 0.0001 by Student’s *t* test. Each data point represents the average value of a single mouse from each group.

First, we validated that proliferating human podocytes are completely differentiated by culturing them at nonpermissive temperature (37 °C) and expressing podocyte-specific markers, such as podocin and nephrin (Supplementary Fig. 1A–D). We validated the expression of GH receptor (GHR) in podocytes, mouse, and human glomeruli (Supplementary Fig. 1E–H). Considering the established role of GH and TGF-β1 in eliciting podocyte injury, we investigated the direct action of GH on the TGF-β/SMAD pathway. TGF-β1 mRNA (Fig. 1A, B) and protein (Fig. 1C, D) levels were upregulated by GH in both dose (0–500 ng/ml) and time-dependent (0–48 h) manner in podocytes. Immunofluorescence analysis also revealed GH-induced TGFβ1 expression in podocytes (Fig. 1E). In addition to TGF-β1, we observed GH-dependent expression of pSTAT3 (Tyr705) and key components of TGF-β1 signaling, such as TGFBR1 (TGF-β receptor 1) and pSMAD2/3 in podocytes (Fig. 1C, D) and HepG2 cells (Supplementary Fig. 2A, B). Analysis of our microarray data (#GEO-GSE21327) revealed the upregulation of TGF-β1 and its receptor in GH-treated podocytes (Supplementary Fig. 2C). As TGF-β1 is a secretory molecule, we measured its levels in conditioned medium from GH-treated podocytes (GH-CM), and observed that GH induces TGF-β1 in both dose and time-dependent manner (Fig. 1F, G). We further verified TGF-β1 activation in GH-treated podocytes by performing a SMAD4 luciferase activity assay (Fig. 1H, I). Furthermore, mouse primary podocytes exposed to GH showed elevated expression of TGFBR1 and its ligand TGF-β1 at both mRNA and protein levels (Fig. 1J–L). TGF-β1 was detected in urine from mice administered with GH (Fig. 1M). To demonstrate the paracrine action of GH-induced TGF-β1, we exposed podocytes and HepG2 cells to conditioned media (CM). GH-CM induced phosphorylation of SMAD2 and 3 in cells naive to GH treatment (Supplementary Fig. 2D, E). Preincubation of GH-CM with neutralizing (anti-GH) antibody (GH-CM + Ab) still able to induce phosphorylation of SMAD2/3. However, exposure of podocytes/HepG2 cells with GH-CM preincubated with anti-GH antibody and supplemented with TGFBR1 inhibitor (SB431542; GH-CM + Ab+SB) could not elicit the phosphorylation of SMAD2/3 (Supplementary Fig. 2F), suggesting that the observed SMAD2/3 activation could be due to TGF-β1 present in GH-CM.

Activated SMAD2 and 3 form a heteromeric complex with SMAD4 and accumulate in the nucleus, following TGF-β1 stimulation and transduce TGF-β signaling. Then, we observed the accumulation of SMAD4 in the nucleus and its enhanced promoter activity (luciferase assay), following treatment with GH-CM or TGF-β1 (Supplementary Fig. 3A). Interestingly, supplementation of GH-CM with SB431542 (GH-CM + SB) abolished the nuclear accumulation of SMAD4 and SMAD4 promoter activity (Supplementary Fig. 3A, B). It is noteworthy that preincubation of GH-CM with anti-GH antibody can induce SMAD4 promoter activity (Supplementary Fig. 3B). Furthermore, the paracrine activity of TGF-β1 in GH-CM was demonstrated using a Cignal SMAD4-GFP reporter assay. GH-CM induced GFP expression in this model, whereas GH-CM preincubated with anti-TGF-β1 antibody failed to elicit GFP expression (Supplementary Fig. 3C). Together, these data support the GH-dependent activation of cognate TGF-β1/SMAD signaling in podocytes.

### TGF-β1 signaling is required for GH-induced Notch reactivation in podocytes

**Fig. 2:**
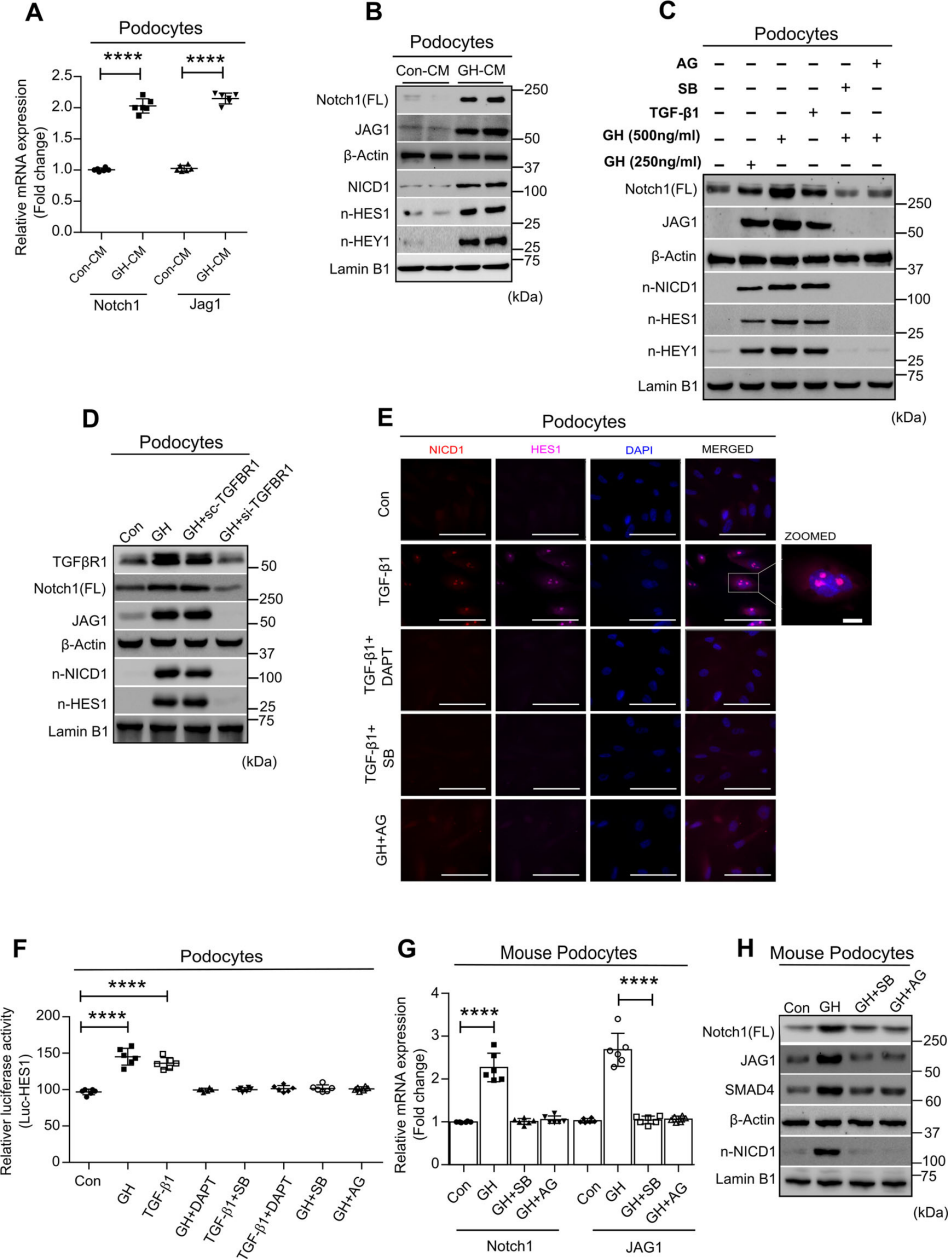
GH induces TGF-β/SMAD pathway-mediated Notch signaling in podocytes. **A** qRT-PCR analysis showing the expression of Notch1 and Jag1 in human podocytes treated with or without a conditioned medium (CM; 50%) from podocytes treated with or without GH for 48 h. Mean ± SD. (*n* = 6). ****p < 0.0001 by Student’s *t* test. mRNA levels were normalized to β-actin and presented as fold change on the *y*-axis. **B** Immunoblotting analysis of podocytes treated with CM (50%) from podocytes treated with or without GH for 48 h. (*n* = 3). **C** Immunoblotting analysis for indicated genes in podocytes treated with or without GH (250 and 500 ng/ml), TGFβ-1 (5 ng/ml), GH (500 ng/ml) + SB431542 (100 nM/ml), and GH (500 ng/ml) + AG490 (10 μM/ml) for 48 h. (*n* = 3). n-NICD1 (nuclear-NICD1), n-HES1 (nuclear HES1), and n-HEY1(nuclear HEY1). **D** Podocytes transfected with siRNA targeting TGFBR1 or scramble RNA (Scr-RNA) were subjected to immunoblotting for indicated genes. (*n* = 3). **E** Immunofluorescence for the nuclear colocalization of NICD1 (red color), HES1 (purple color), and counterstained with DAPI (blue color) in podocytes treated with or without GH for 48 h. Magnification ×630. Scale bar = 20 μm. (*n* = 3). **F** HES1 reporter activity was measured in podocytes exposed to GH for 48 h. Mean ± SD. **G** qRT-PCR analysis for Notch1 and JAG1 in podocytes isolated from a mouse treated with or without GH (1.5 mg/kg b.w), GH + SB431542 (1 mg/kg b.w), and GH + AG490 (1 mg/kg b.w). Mean ± SD. (*n* = 6). **F**, **G** ****p < 0.0001 by one-way ANOVA post hoc Dunnett test (*n* = 6). **H** Representative immunoblots for indicated genes in podocytes isolated from mice treated with or without GH. (*n* = 3). β-Actin and lamin-B1 served as an internal control.

Notch activation was undetectable in glomeruli from healthy adult kidneys, unlike their progenitors in the fetal kidney, which show enhanced Notch activity^15^. Recently, we showed that GH activates Notch signaling in adult podocytes^9^. Niranjan et al. reported that TGF-β1 induces Notch in podocytes from diabetic mice^16^. Since circulating GH levels are elevated in poorly controlled type 1 diabetes milieu, and both GH and TGF-β1 were shown to induce Notch signaling, we sought to investigate whether GH activates Notch signaling via TGF-β1. When podocytes were exposed to GH or GH-CM, elevated expression of Notch signaling components (Notch, its ligand Jag1; Notch intracellular domain (NICD1); Notch targets, HES1 and HEY1) was observed (Fig. 2A, B vs 2C). Interestingly, expression of Notch signaling components was ameliorated when podocytes were treated with GH in the presence of inhibitors for either JAK2 (AG490) or TGFBR1 (SB431542; Fig. 2C). GH-CM’s ability to induce nuclear localization of HES1 and HEY1 in podocytes was impaired when supplemented with TGFBR1 inhibitor, SB431542 (Supplementary Fig. 3D). To elucidate the essential role of TGFBR1 in GH-induced Notch activation, we engineered podocytes with siRNA-mediated knockdown of TGFBR1. GH was unable to induced Notch activation in podocytes with reduced TGFBR1 expression (Fig. 2D). The transcriptional activity of NICD1 was determined by both its nuclear localization (Fig. 2E) and HES1-luciferase reporter assay (Fig. 2F) was found to be elevated upon GH or TGF-β1 treatment. GH- or TGF-β1-induced nuclear localization of NICD in podocytes was abolished in the presence of AG490 or SB431542 (Fig. 2E). Similarly, the observed increase (~45%) in the transcriptional activity of HES1 by GH or TGF-β1 was compromised in the presence of AG490 or SB431542 (Fig. 2F). γ-Secretase is an intracellular protease that cleaves NICD from the Notch receptor and triggers the Notch signaling cascade. Increased γ-secretase activity by GH and TGF-β1 was abolished when podocytes were treated with AG490 or SB431542 (Supplementary Fig. 3E). Indeed, inhibition of γ-secretase activity by (N-[N-(3,5-difluorophenacetyl)-L-alanyl]-S-phenyl glycine t-butyl ester (DAPT) abolished HES1 promoter activity in podocytes exposed to GH or TGF-β1 (Fig. 2F). Notch activation in podocytes isolated from GH-administered mice was also ameliorated in the presence of SB431542 or AG490, as assessed by qRT-PCR and immunoblotting (Fig. 2G, H). Together, these data suggest that GH activates Notch signaling in podocytes via a TGF-β1/TGFBR1 axis.

### Both GH and TGF-β1 induce cell cycle reentry of quiescent podocytes in Notch1-dependent manner

**Fig. 3:**
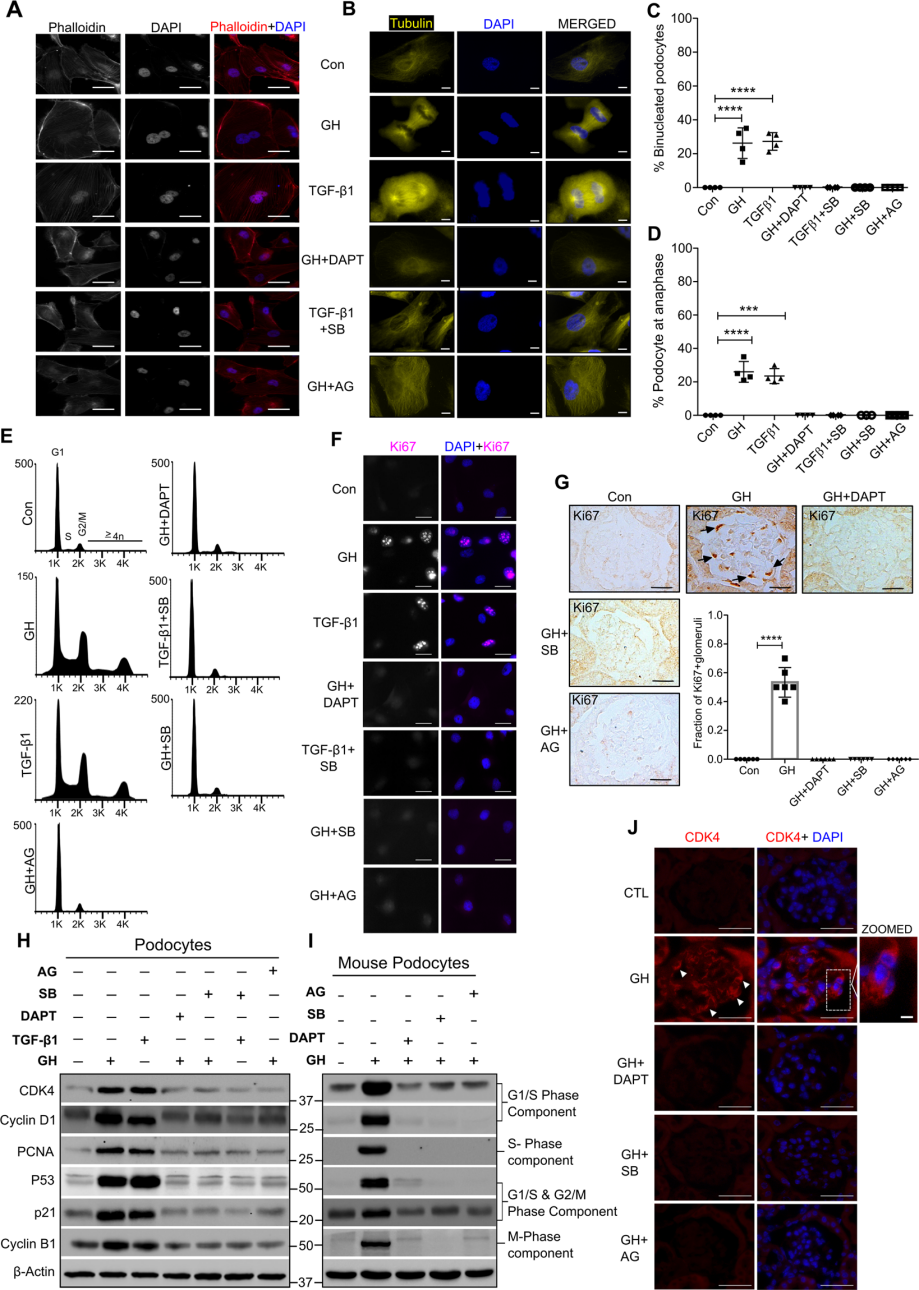
GH stimulates cell cycle reentry and binucleation in podocytes. **A**, **B** Representative images of F-actin (green color), α-tubulin (yellow color), and counterstained with DAPI (blue color) staining in human podocytes treated with or without treatment for 48 h. Magnification ×630. Scale bar = 20 μm. (*n* = 3). **C** The graph represents the % of binucleated podocytes from the above experimental conditions. Mean ± SD. (*n* = 4). Each data point represents the average value of fifty cells. **D** The graph represents the % of podocytes accumulated in anaphase from the above experimental conditions. Mean ± SD. (*n* = 4). **C**, **D** ***p < 0.001 and ****p < 0.0001 by one-way ANOVA post hoc Dunnett test. Each data point represents the average value of fifty cells. **E** Cell cycle phases of podocytes from indicated experimental conditions (*n* = 4). **F** Immunofluorescence for the Ki67 (red color) and counterstained with DAPI (blue color). Magnification ×630. Scale bar = 20 μm. (*n* = 3). **G** Representative images of immunohistochemistry for anti-Ki67 expression by DAB staining in mice glomerular sections. Quantification of Ki67-postive glomeruli (right panel) where each dot represents the average value of ten glomeruli from each mouse. Black arrow indicates specific expression of Ki67 in podocytes. Magnification ×630. Scale bar = 20 μm. (*n* = 3). ****p < 0.0001 by one-way ANOVA post hoc Dunnett test. **H**, **I** Immunoblotting analysis in human podocytes and mice primary podocytes. (*n* = 3). **J** Representative images of immunostaining for CDK4 (red color) and counterstained with DAPI (blue color) in mice glomeruli. Magnification ×630. Scale bar = 20 μm. (*n* = 3). White arrowhead indicates specific expression of CDK4 in the podocyte.

In healthy mice and human, mature podocytes are in the quiescent stage (G0 phase), a prerequisite for highly specialized functions^17^. Mature podocytes are differentiated and express high levels of cyclin-dependent kinase (CDK) inhibitors, suggesting that they lack the ability to renew during adult life^18^. CyclinB1, the regulatory subunit of CDK1 and essential molecule to induce cell proliferation, was found to be upregulated in podocytes exposed to GH (Supplementary Fig. 4A). Live-cell imaging confirmed that GH-treated podocytes were accumulated in anaphase (Supplementary Movies 1 and 2). Since Notch signaling was shown to induce proliferation of embryonic stem cells and cell cycle reentry of terminally differentiated cells^19,20^, we assessed whether activated Notch signaling induces cell cycle reentry of podocytes in our experimental model. Morphological screening by phalloidin staining revealed 24 ± 7% of GH- or TGF-β1-treated podocytes were binucleated and hypertrophic (Fig. 3A, C). Interestingly, DAPT selectively mitigated GH-induced podocyte binucleation compared to hypertrophy (Fig. 3A, C). Alternatively, we found that 27 ± 10% of GH- or TGF-β1-treated podocytes were in anaphase as suggested by microtubule formation (Fig. 3B, D), whereas DAPT prevented GH-induced cell cycle progression (Fig. 3B, D).

We performed flow cytometric analysis to ascertain the activation of cell cycle events with GH or TGF-β1 (Fig. 3E). Flow cytometry data revealed that ~40% and 31% of GH-treated podocytes were in S and G2/M phase, respectively (Fig. 3E). Similarly, ~35% and 28% of TGF-β1-treated podocytes were also accumulated in the S and G2/M phase, respectively (Fig. 3E). As anticipated, AG490 and SB431542 abrogated GH and TGF-β1-induced cell cycle reentry. We observed elevated Ki67 expression strongly associated with cell proliferation in GH-treated podocytes in vitro (Fig. 3F) and in vivo (Fig. 3G). Despite podocytes displaying proliferative phenotype when exposed to GH and TGF-β1, they also showed binucleation, suggesting only successful karyokinesis, but not cytokinesis. Therefore, we assessed the expression of proliferating markers (PCNA), cell cycle regulators, and checkpoints. Interestingly, in addition to cell cycle activators (CDK4 and cyclinD1), we also observed elevated expression of both G1/S and G2/M checkpoints, implying complex two-tier regulation cell cycle events in podocytes exposed to GH or TGF-β1 (Fig. 3H–J). Inhibition of JAK2 or TGFBR1 mitigated Ki67 expression (Fig. 3F, G) and attenuated cell cycle regulators in both human and mouse podocytes (Fig. 3H–J). Strikingly, inhibition of Notch activation by DAPT abrogated GH-induced proliferating markers and, in turn, activation of cell cycle events (Fig. 3F–J). Together, these data reveal that podocytes overcome the quiescence and reenter the cell cycle during stimuli, such as exposure to GH or TGF-β1 in a Notch1-dependent manner, whereas inhibition of Notch activation prevented cell cycle reentry.

### Cytokinesis failure induces apoptosis in GH- or TGF-β1-treated podocytes

**Fig. 4:**
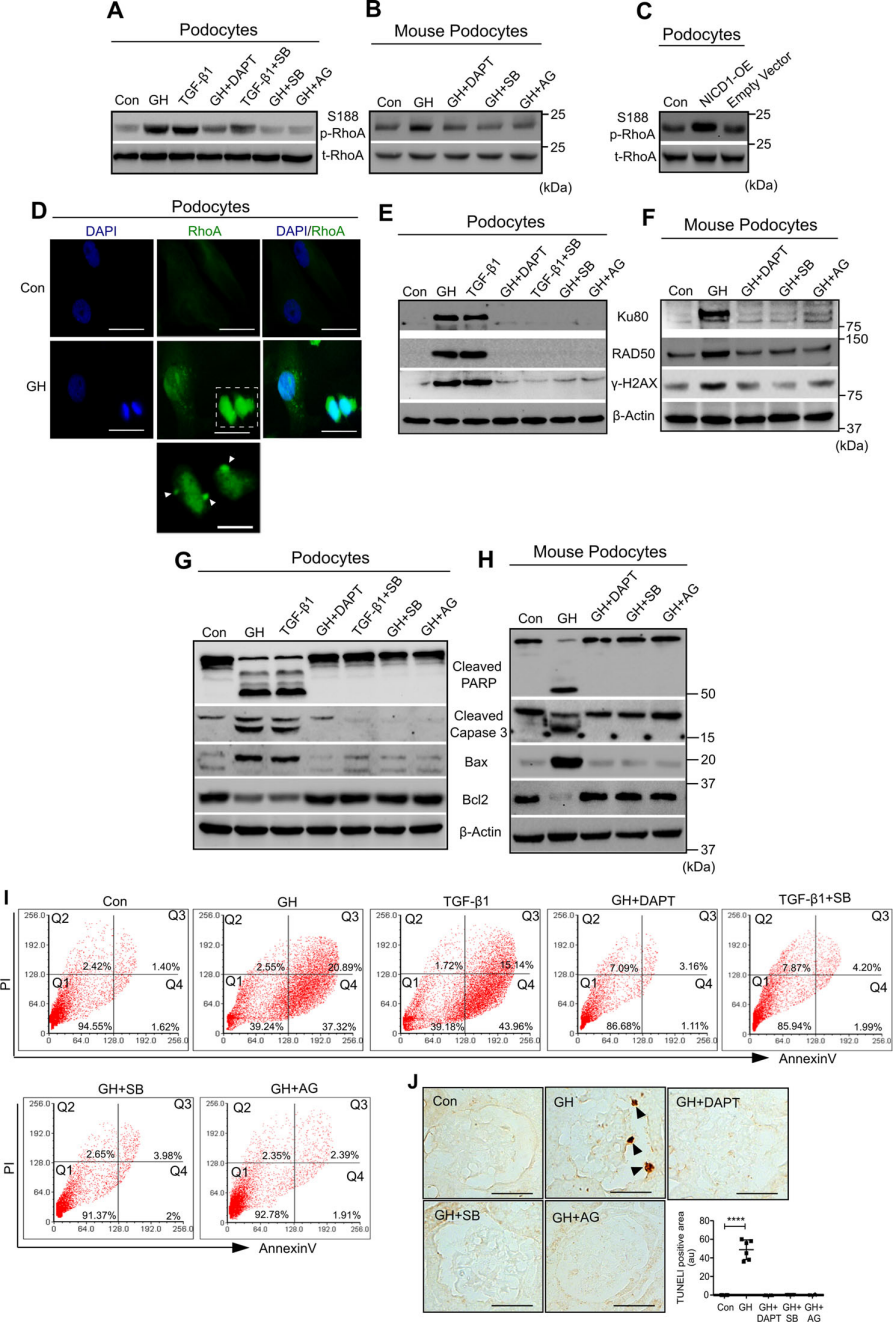
GH-induced TGF-β leads to podocyte DNA damage and apoptosis. **A**, **B** Immunoblotting analysis in human podocytes (treated for 48 h) and from mouse podocytes (*n* = 3). **C** Immunoblotting analysis in human podocytes ectopically expressing NICD1 (NICD1-OE). (*n* = 3). **D** Immunofluorescence for the RhoA (green color) and counterstained with DAPI (blue color) in human podocytes treated with GH for 48 h. Magnification ×630. Scale bar = 20 μm. (*n* = 3). White arrowhead indicates the delocalization of RhoA from midbody. **E**–**H** Immunoblotting analysis in human podocytes (**E**, **G**) and mouse podocytes (**F**, **H**). (*n* = 3). **I** Human podocytes stained with FITC-Annexin V and PI and analyzed by flow cytometry. (*n* = 4). The values of the representative histograms indicate the percentage of podocytes in the lower left quadrant (live cells), lower right quadrant (early apoptosis), upper right quadrant (late apoptosis), and upper left quadrant (necrotic cells). (*n* = 3). **J** Representative TUNEL (terminal deoxynucleotidyl transferase dUTP nick-end labeling) staining by DAB in mice glomerular sections. Quantification of TUNEL-postive glomeruli (right panel), where each dot represents the average value of ten glomeruli from each mouse. Magnification ×630, Scale bar = 20 μm. (*n* = 3). ****p < 0.0001 by one-way ANOVA post hoc Dunnett test. β-Actin served as an internal control.

One of the mechanisms for binucleation could be incomplete cytokinesis due to aberration in contractile ring assembly or the ingression phase of cytokinesis. Live-cell imaging suggested incomplete cytokinesis in GH-treated podocytes (Supplementary Fig. 4B). RhoA, a member of the Rho GTPase family, is essential for cytokinesis via acting midbody during cleavage furrow ingression and successful generation of two daughter cells^21,22^. Elevated RhoA expression was observed in podocytes either treated with GH or TGF-β1 (Fig. 4A, B), and during ectopic expression of NICD1 (Fig. 4C). On the other hand, inhibition of Notch reduced GH- or TGF-β1-induced RhoA expression (Fig. 4B). Interestingly, abnormal localization of RhoA (distant to the contractile ring) was observed in podocytes treated with GH (Fig. 4D).

Although GH enforced quiescent podocytes to undergo cell cycle reentry, these cells failed to accomplish successful cell division. Often, the failure of cell cycle progression is accompanied by induction of cell death. Live-cell imaging suggested incomplete cytokinesis in GH-treated podocytes and apoptosis (Supplementary Fig. 4C and Supplementary Movie 3). Therefore, investigated markers of DNA damage and apoptosis in podocytes exposed to GH or TGF-β1. Elevated expression of γ-H2X (a marker for DNA double-strand break), Ku80, and Rad50 (markers for double-strand repair) was observed in GH, and TGF-β1-treated human podocytes and also in podocytes isolated from mice administered with GH (Fig. 4E, F). The increased levels of cleaved forms of PARP and caspase-3, induction of Bax (proapoptotic markers), and suppression of Bcl2 were observed in podocytes exposed to GH or TGF-β1 (Fig. 4G, H and Supplementary Fig. 5A, B). Interestingly, GH- or TGF-β1-induced DNA damage (Fig. 4E, F) and apoptotic (Fig. 4G, H) markers were ameliorated by DAPT and AG490 or SB431542.

The majority of GH- or TGF-β1-treated podocytes are early apoptotic (40 ± 5% vs late 15 ± 7%; Fig. 4I). Caspase-3 stainings also revealed podocyte apoptosis with characteristic blebbing of the cell body (Supplementary Fig. 5C). However, DAPT, SB431542, and AG490 prevented podocyte apoptosis (Fig. 4I and Supplementary Fig. 5C). Similarly, TUNEL staining also showed increased podocyte apoptosis in GH-treated mice, whereas DAPT, SB431542, and AG490 treatment ameliorated GH-induced apoptosis (Fig. 4J). Our data suggest GH or TGFβ1 treatment, despite inducing mitosis activation, evoked cell death, a phenomenon is known as mitotic catastrophe^23^. Results described above indicated that exposure of podocytes to GH-induced mitotic catastrophe-associated cell death. We further ascertained the GHR/JAK2 axis’s essential role in podocyte injury with GHR expression knockdown. In podocytes transfected with GHR siRNA, GH failed to induce phosphorylation of JAK2, STAT3, SMADs, and expression of NICD1 (Supplementary Fig. 5D). Knockdown of GHR expression resulted in the blunting of the ability of GH to elicit caspase-3 activation and cyclinB1 expression and phosphorylation of RhoA (Supplementary Fig. 5D). Hence, these results indicate that GH transduces the observed effects via GHR/JAK2 axis. Podocyte-specific deletion of GHR (pGHR conditional knockout mice, pGHRKO) protected these mice from streptozotocin-induced decline in kidney function as evidenced by an improved urinary albumin–creatin ratio (UACR) and proteinuria (Supplementary Fig. 5E, F). Conditional deletion of podocyte GHR prevented Notch activation as evidenced by decreased NICD1 expression compared with heterozygous (pGHR+/−) mice in the diabetic settings (Supplementary Fig. 5G).

### Blocking of TGFBR1 or Notch1 signaling abrogates GH-induced podocytopathy and proteinuria

**Fig. 5:**
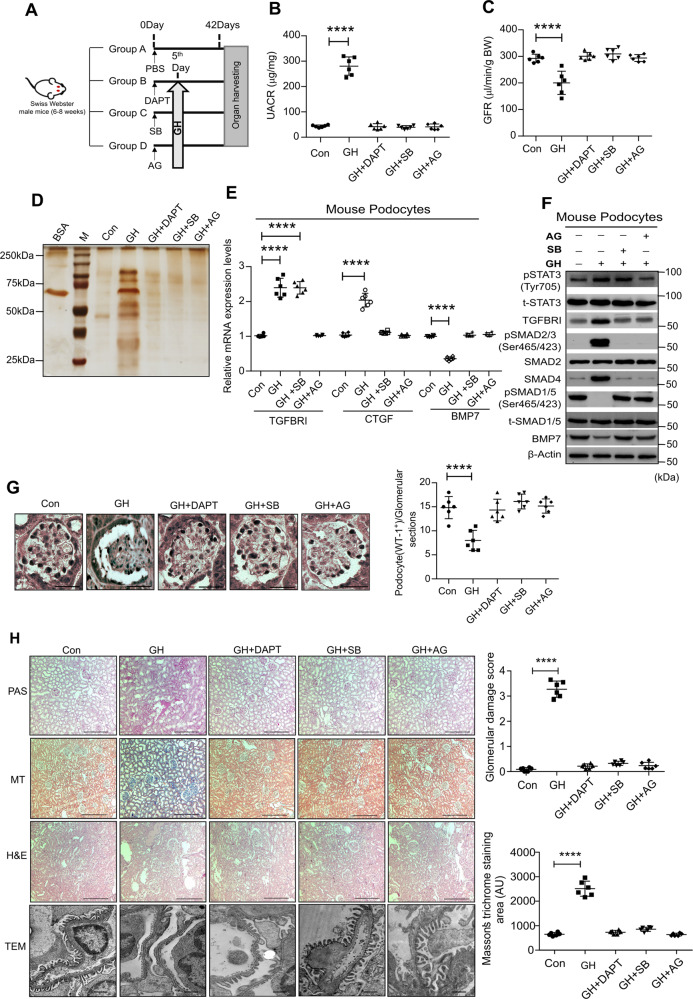
Blockade of GHR and TGFβR1 protects mice from GH-induced proteinuria. **A** Schematic presentation of mouse experimentation. **B** Urinary albumin creatinine ratio (UACR) and **C** glomerular filtration rate (GFR) was estimated in the experimental mice. Mean ± SD. ****p < 0.0001 by one-way ANOVA post hoc Dunnett test. Each dot represents the average value of three independent experiments from a single mouse. **D** Silver staining was performed to the urine samples from the mice. *n* = 3. BSA bovine serum albumin, M protein standard marker. **E** qRT-PCR analysis in mouse podocytes from control and treatment groups. Mean ± SD. ****p < 0.0001 by one-way ANOVA post hoc Dunnett test. Each data point represents the average value of three independent analysis from each mouse (*n* = 6). **F** Immunoblotting analysis in mouse podocytes from control and treatment groups. (*n* = 3). **G** Representative images of immunohistochemical staining for anti-WT1 (podocytes) by DAB in the glomerulus sections from control and treatment groups. Magnification ×630, Scale bar = 20 μm. (*n* = 3). The average number of WT1 + cells (right panel) in the mice glomerulus was quantified using ImageJ (NIH). Mean ± SD. ****p < 0.0001 by one-way ANOVA post hoc Dunnett test. Each data point represents the average value of three independent experiments from each mouse (*n* = 6). **H** Representative image of PAS (periodic acid–Schiff), MT (Masson’s trichrome), and H&E (hematoxylin and eosin) staining of glomeruli, and TEM (transmission electron microscopy) imaging of podocytes. Magnification ×100. Scale bar = 100 μm, TEM scale bar 1 μm. Glomerular damage score and MT-stained area (right panel) were quantified using ImageJ (NIH) and prepesnted as a dot plot, where each dot represents the average value of ten glomeruli from each mouse (*n* = 6). Mean ± SD. ****p < 0.0001 by one-way ANOVA post hoc Dunnett test.

Administration of GH to mice resulted in increased UACR and proteinuria and a decline in glomerular filtration rate (GFR; Fig. 5A–D). Podocytes isolated from GH-treated mice showed elevated levels of TGBR1 and connective tissue growth factor (CTGF), and a decreased level of bone morphogenetic protein 7 (BMP-7; Fig. 5E, F). CTGF is a TGF-β1 target, whereas BMP-7 is an antagonist of TGF-β1. Furthermore, we also noticed the activation of canonical SMAD signaling in podocytes isolated from GH-treated mice (Fig. 5F). As expected, blocking GHR (by AG490) or TGBR1 (by SB431542) prevented activation of SMAD signaling in GH-administered mice (Fig. 5F). Since GH-treated podocytes eventually undergo apoptosis in vitro, to ascertain in vivo confirmation, we counted the number of podocytes per glomerulus in mice administered with GH. The number of podocytes (WT1 positive) in GH-treated mice decreased significantly (*p* < 0.005) compared with mice naive to GH treatment (Fig. 5G, left and right panel). Blunting the Notch activation, inhibition of JAK2 or TGFBR1 mitigated GH-induced podocyte depletion as determined by WT1 staining (Fig. 5G). Expression of SD proteins (podocin and ZO-1) was decreased in podocytes from GH-administred mice (Supplementary Fig. 6A). Since podocyte loss and damage to the SD eventually manifest in glomerulosclerosis in addition to proteinuria^24,25^, we measured the histopathological changes by periodic acid–Schiff (PAS), Masson’s trichrome (MT), hematoxylin and eosin (H&E) staining, and transmission electron microscope (TEM) imaging. Significant glomerulosclerosis (PAS and MT staining) and altered morphology (H&E staining) were observed in GH-treated mice (Fig. 5H). TEM images revealed podocyte foot process effacement and thickening of the GBM (Fig. 5H). Blunting GH or TGF-β1 action or blocking Notch activation protected from GH-mediated adverse histological manifestations (Fig. 5H). Suppression of GH or TGF-β1 action or preventing Notch signaling preserved the expression of podocyte SD proteins (podocin and ZO-1; Supplementary Fig. 6A). Blunting GH or TGF-β1 action ameliorated TGF-β1 loss into the urine and the extent of glomerulosclerosis as evidenced by MT-stained positive area (Supplementary Fig. 6B, C). Together, our data demonstrate that TGF-β1 mediates GH’s role in the pathogenesis of nephropathy.

### Hyperactivated Notch signaling and binucleated podocytes in patients with diabetic nephropathy

**Fig. 6:**
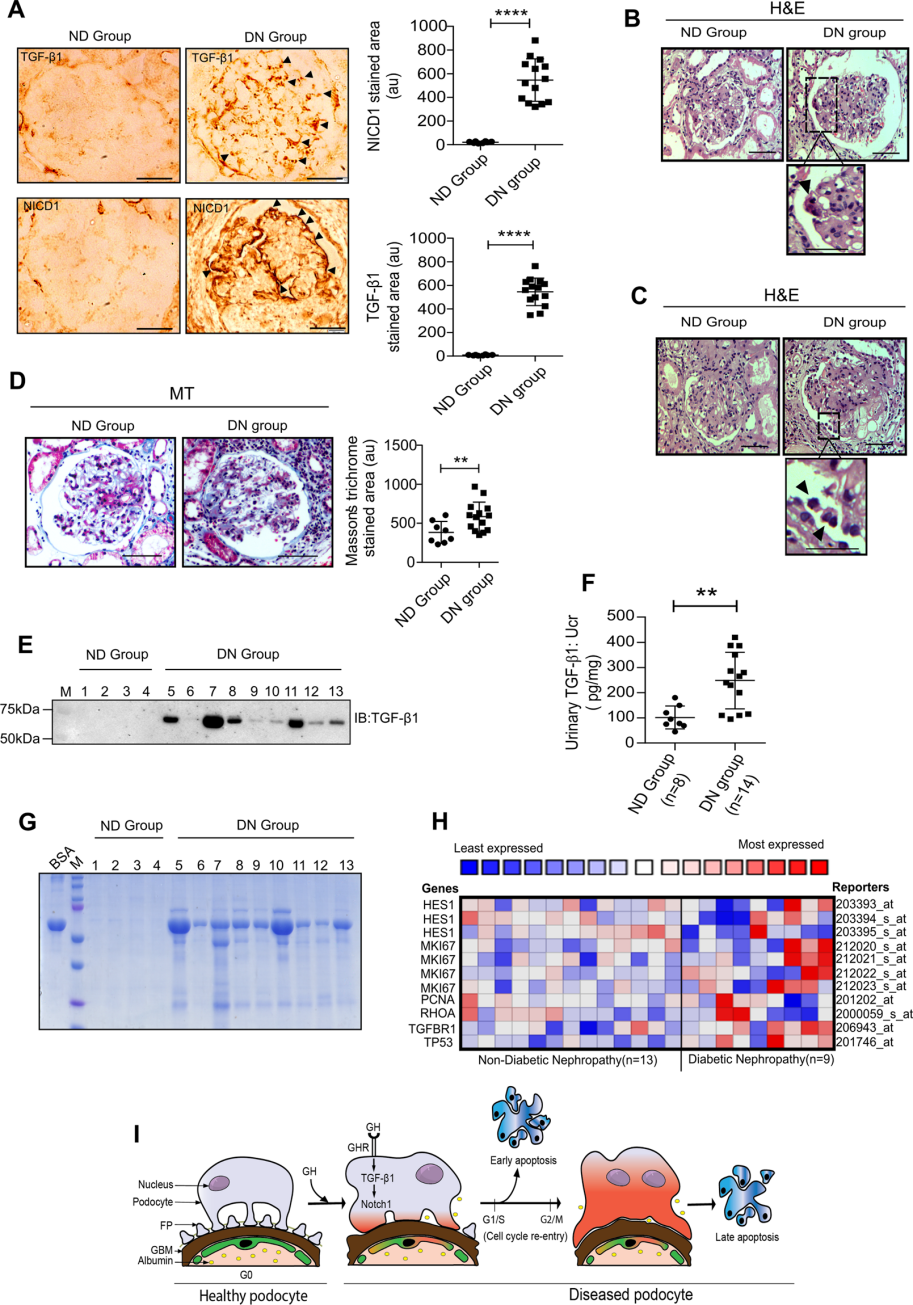
Elevated TGF-β1 signaling and proteinuria correlated in people with DN. **A** Representative images of immunohistochemical staining for TGFβ-1 and NICD1 by DAB in the glomerulus sections from nondiabetic (ND; *n* = 8) and diabetic nephropathy (DN; *n* = 14) patients. Magnification ×630. Scale bar = 20 μm. (*n* = 3). NICD1 and TGF-β1-stained area in glomerulus (right panel) were quantified using ImageJ (NIH) and presented as a dot plot. Mean ± SD. ****p < 0.0001 by Student’s *t* test. **B**, **C** Representative image of H&E stain in glomerular sections from ND and DN groups. Black arrowhead indicates binucleated and detached podocyte. Magnification ×630. Scale bar = 20 μm. Zoomed picture emphasizes a binucleated and detached podocyte. **D** Representative image of MT stain in glomerular sections from ND and DN groups. Magnification ×630. Scale bar = 20 μm. MT-stained area in glomerulus was quantified using ImageJ (NIH) and presented as a dot plot. Mean ± SD. ****p < 0.0001 by Student’s *t* test. **E** Immunoblotting analysis for TGF-β1 in the urine samples from ND (*n* = 4) and DN groups (*n* = 9). IB immunoblot. **F** Quantification of TGF-β1 in the urine samples from ND (*n* = 8) and DN (*n* = 14) groups. Mean ± SD. **p < 0.001 by Student’s *t* test. **G** Urine samples from ND (*n* = 4) and DN (*n* = 9) were resolved on SDS–PAGE and stained with Coomassie Blue. BSA bovine serum albumin, M protein standard marker. **H** Nephroseq (www.nephroseq.org) analysis comparing HES1, MIKI67, PCNA, RHOA, TGFBR1, and TP53 expression levels in ND (*n* = 13) versus DN (*N* = 9) in Woroniecka diabetes of glomerular tissue. Data indicate that the expression of these genes increased >1.5-fold in the DN. **I** Schematic illustration of GH action on podocyte cell cycle entry and apoptosis via TGF-β1-mediated Notch1 activation.

We evaluated the extent of NICD1 expression and binucleation of podocytes in subjects with DN. Kidney biopsy sections from subjects with diabetes showed increased TGF-β1 and active Notch (NICD1) expression (Fig. 6A). We observed binucleated podocytes (Fig. 6B) and detached podocytes localized to urinary space in glomeruli from DN subjects (Fig. 6C). As anticipated, glomerulosclerosis was observed in kidney sections from subjects with DN (Fig. 6D). We have also observed elevated urinary TGF-β1 levels from these subjects with DN (Fig. 6E, F). As expected, subjects with diabetes showed severe proteinuria (Fig. 6G). Interestingly, Nephroseq (https://www.nephroseq.org) analysis revealed co-expression of TGFBR1, Notch signaling components (HES1), cell proliferating markers (Ki67 and PCNA), cell cycle regulator (TP53), and regulator of cytokinesis (RhoA) in human diabetes glomerulus dataset (Woroniecka; Fig. 6H). These data confirm that in DN, there is elevated functional Notch signaling in their glomeruli, podocytes with aberrant cell cycle entry, and enhanced podocyte injury markers.

## Discussion

The current investigation reveals a novel mechanism for GH action on glomerular podocytes and pathogenesis of DN. The major findings of this study in podocytes are, GH induces TGF-β1, activates canonical TGF-β1/SMAD signaling pathways, which in turn activate Notch signaling. Notch activation is implicated in the cell cycle reentry of podocytes. However, these activated podocytes fail to complete the mitotic cycle, and as a consequence, binucleated podocytes are accumulated (Fig. 6I). Indeed, renal biopsies from patients with DN also revealed binucleated podocytes. Podocytes exposed to GH or TGF-β1 fail to accomplish mitosis due to cytokinesis failure and are susceptible to apoptosis. Importantly, inhibition of JAK2 and TGFBR1 successfully protected mouse podocytes from GH- and TGF-β1-induced Notch activation, cell cycle reentry, and apoptosis. We observed podocyte loss, glomerulosclerosis, and proteinuria in GH-treated mice, common DN features. The current study demonstrates that the TGF-β1/SMAD signaling mediates GH’s role in DN. Interestingly, inhibition of Notch with DAPT significantly ameliorated both GH- and TGF-β1-induced podocyte injury and apoptosis.

The first important observation from our study was that GH induces TGF-β1 expression in podocytes. Elevated GH levels are implicated in the early renal hypertrophy, depletion of podocytes, and proteinuria in poorly controlled T1DM^26,27^. However, it was not clear whether this causal role of the GH in the pathogenesis of diabetic kidney disease is due to direct actions of GH on the podocytes or indirect via effector molecules and/or pathways. Among several hosts of mediators in the diabetic milieu, TGF-β1 has emerged to have a key role in the development of morphologic manifestations and clinical characteristics of DN^22,28–30^. Inhibition of TGF-β1 or ablation of SMAD3 (SMAD^−/−^) showed promising protection from glomerulosclerosis and renal dysfunction^31^. Despite knowing that activation of TGF-β/SMAD signaling is crucial in most kidney diseases, the systemic stimuli that activate this pathway in the podocyte remain unclear. Previous studies proposed that high glucose and angiotensin II induces TGF-β1 expression in mesangial cells; however, in the podocytes, angiotensin II does alter TGF-β expression^30,32^. Although, elevated GH levels and overactivity of the GH/GHR axis are implicated in renal manifestations and chronic kidney disease^33^, the temporal association between GH and TGF-β is unclear. It is noteworthy that GH-induced mild glomerulosclerosis and interstitial fibrosis in diabetic Sprague–Dawley rats is associated with an elevation in TGF-β1 levels^12^ and suppressing JAK2, an immediate downstream target of GH reduced TGF-β mRNA expression^13^. In the present study, we establish that GH stimulates TGF-β1 in podocytes, and to the best of our knowledge, this is the first study to demonstrate that GH induces TGF-β1 expression in any cell type.

Another key finding that emerged from our study is that both GH and TGF-β1 trigger cell cycle reentry of podocytes by Notch activation. The Notch is a highly conserved juxtracrine signaling cascade, which transduces short-range signals between neighboring cells. This pathway comprises four transmembrane receptors (Notch1–4) and five ligands (Jagged 1 and 2; Delta-like 1, 3, and 4). Binding of ligand to Notch receptor results in shedding of both the Notch extracellular domain by ADAM protease and cleavage of the NICD by the γ-secretase complex. Subsequent nuclear translocation of NICD activates the expression of target genes, such as Hes1 and Hey1. We had previously demonstrated that GH induces Notch activation^9^. Since TGF-β1 is a powerful Notch activator, we investigated whether the observed GH-dependent secretion of Notch activation could be mediated via GH-dependent secretion of TGF-β1. Although active Notch signaling is required until the S-shaped body formation stage during nephrogenesis, it is almost undetectable in healthy adult glomeruli. Indeed, the Notch pathway’s downregulation is required for renal progenitors to differentiate toward podocyte lineage^15^. Mature podocytes exit from the cell cycle evidenced by reduced expression of proliferating markers (e.g., Ki67, PCNA, and cyclinB1) and increased expression of cell cycle inhibitors p57 and p27^34^. The persistent expression of p57 and p27 enables the mature podocytes to remain quiescent^35,36^. Our data reveal that GH or TGF-β1 stimulated senescent podocytes to reenter the cell cycle and progressed to the S- and G2/M phase. The progression of cell cycle events is concomitant with the activation of Notch signaling. Notch activation stimulates cell division in renal progenitors, whereas in differentiated podocytes, it helps cells overcome the G2/M checkpoint^15^. Increased Notch activity was observed in podocytes of patients with glomerular disorders, while blunting Notch activity ameliorated glomerulosclerosis, prevented podocyte death during the initial phases of glomerular injury, and proteinuria ^16^.

A significant observation from this study is that podocytes exposed to GH become binucleated and hypertrophic. Normally, postmitotic cells do not reenter the cell cycle when exposed to growth signals. Stressed podocytes reenter the cell cycle and are arrested at the G2/M restriction point by CDK inhibitors, and become hypertrophic^37^. Multi-nucleation of podocytes contributes to the increased cell size. The hypertrophic phenotype of podocytes appears to be transient as podocytes with cytokinesis failure and aneuploidy are susceptible to cell death^23^. Podocyte depletion has been considered as a hallmark of both primary and secondary forms of glomerulosclerosis^38–40^. Decreased podocyte density in the early stage of diabetic patients and experimental animal models, which strongly correlates with the severity of proteinuria and DN progression^41–43^. A large body of evidence suggests podocytes undergo apoptosis, which is considered as a major form of podocyte loss that culminates in severe glomerular injury^44,45^, EMT of podocytes is considered an alternative cause for podocyte loss^46^. We have noticed both early and late phase apoptosis. Cell cycle transition from G1 to S phase leads to extensive DNA damage that culminates in the early apoptosis of podocytes. In comparison, late apoptosis suggests that the podocytes with DNA damage that overcome the G2/M checkpoint eventually failed to complete cytokinesis. These cells accumulated at the G2/M phase of the cell cycle with increased DNA content per cell and eventually undergo late apoptosis. Most of the podocytes in our experiments underwent early apoptosis in response to GH treatment, suggesting that these terminally differentiated podocytes are not sufficiently competent to carry cell cycle events successfully despite mitogenic stimuli by GH or TGF-β1. Wu et al. reported that TGF-β at a lower concentration promotes podocyte differentiation, whereas TGF-β levels beyond a critical threshold induce G2/M block and apoptosis^47^. Whereas GH plays a significant role in normal renal function, overactive GH signaling has been implicated in proteinuria in diabetes. Together these studies suggest in a dose-dependent manner, GH and TGF-β specify podocyte fate.

Mature podocytes possess high cytoplasm to nucleus ratio and express highly organized myofibrils that normally prevent cell division. Disassembly of cytoskeletal filaments during mitogenic stimuli would adversely affect their function. Another interesting feature of differentiated podocytes is that they express a wide range of cell cycle proteins, which could be a prerequisite for executing mitotic catastrophe in response to stress signals, such as GH and TGF-β1. The mechanisms by which cell cycle reentry causes cell death are not completely explained. Still, cytokinesis failure and abnormal Rho distribution observed in our study could be one of the major reasons. Further studies are required to delineate the contribution of specific STATs in GH-mediated TGF-β1 production and whether the source of GH in vivo is more local than endocrine or opposite. Based on our observations, we propose that GH induces TGF-β1 expression and is a causative factor in developing podocyte hypertrophy, podocyte injury, and consequent proteinuria during GH-induced kidney diseases. In summary, the present report establishes that GH induces TGF-β1 expression in podocytes. Some of the actions of GH on the podocytes are mediated through TGF-β1 in an autocrine and paracrine manner. Our data provide a mechanistic link between GH and podocyte dysfunction in diseases like type I diabetes mellitus and acromegaly.

## Materials and methods

### Antibodies and reagents

The primary antibodies are as follows: anti-activated Notch1 (ab8925), anti-pSTAT3 (ab76315), anti-STAT3 (ab5073), anti-TGF-βR1 (ab31013), anti-HEY1 (ab154077), anti-p53 (ab26), anti-RhoA (ab54835), anti-cyclinB1 (ab72), anti-p21 (ab109520), anti-α-Tubulin (ab7291), anti-CDK4 (ab137675), anti-Ki67 (ab16667), anti-cyclinD1 (ab16663), and anti-Laminin-B1 (ab16048) were purchased from Abcam (Cambridge, MA). The anti-Notch1-full-length(#3608), anti-cleaved-Notch1 (#4147 S), anti-pSMAD2/3 (#8828), anti-SMAD2 (#5339), anti-pSMAD1/5 (#9516), anti-SMAD5 (#12534), anti-cleaved-PARP (#5625), anti-cleaved caspase-3 (#9664), anti-BMP-7 (#4693), anti-BAX (#89477), anti-Bcl2 (#15071) anti-pJak2 (#3771), anti-Jak2 (#3230), anti-SMAD4 (#46535), and anti-β-actin (#4970) were purchased from Cell Signaling Technology (Danvers, MA). The anti-Ku80 (NBP156408), anti-RAD50 (NB100-147), anti-γ-H2AX (NB100-384), anti-Nephrin (NBP1-77303), anti-podocin (NBP2-75624), and anti-PCNA (NBP500-106). The anti-TGF-β1 (MAB240) from R&D Systems (Minneapolis, MN). The anti-HES1 (sc-166410) anti-WT-1 (sc-393498), anti-GHR (sc-137185), and anti-ZO-1 (sc-33725) were obtained from Santa Cruz Biotechnology (Dallas, TX). Anti-JAG1 (PAB807Hu01) was purchased from Cloud-clone (Houston, TX). Anti-caspase-3 (9H19L2) and anti-TGF-β1 for immunoblotting and immunohistochemistry (MA5-16949) and for immunofluorescence (TA506583) were purchased from Thermo Fischer Scientific (Waltham, MA). Mouse/Rabbit PolyDetector DAB HRP Brown Immunochemistry detection system (BSB020, Santa Barbara, CA). Phalloidin fluorescein isothiocyanate labeled (P5282) and glutaraldehyde solution (G5882) were obtained from Sigma Aldrich (St. Louis, MO, USA). Precision Plus Protein Dual Color Standards (Bio-Rad, Hercules, CA) and ProLongTM Diamond Antifade Mountant (P36961) were purchased from Molecular Probes Life Technologies. DyLight 488 and DyLight 564, and Cy5-conjugated secondary antibody were purchased from Vector Laboratories (Burlingame, CA). Primers used in this study procured from Integrated DNA Technologies (Coralville, IA). Paraformaldehyde (P6148-500G), bovine serum albumin (BSA, A3294-50G), fluorescent phalloidin–TRITC conjugate (P1951), 4, 6-diamidino-2-phenylindole (DAPI, P36971). All other reagents used were of analytical grade and obtained from Sigma Aldrich (St. Louis, MO, USA).

### Experimental drugs

DAPT (#D5942) was purchased from Sigma Aldrich (St. Louis, MO, USA), TGF-βR1 inhibitor (SB431542), and JAK2 inhibitor (AG490) were purchased from Tocris Bioscience (Pune, India). Recombinant TGF-β1 (#240-B-002) was purchased from R&D Systems (Minneapolis, MN), and recombinant GH (Genotropin) was procured from Pfizer (NY).

### Podocyte culture and experimentation

In this study, conditionally immortalized human podocytes (a gift from Prof. Moin Saleem, University of Bristol) cells were cultured essentially as described earlier^9^. Briefly, after 14 days of differentiation at 37 °C, podocytes were treated with or without GH, GH + DAPT, TGF-β1, TGF-β1 + SB431542, GH + SB431542, and GH + AG490. Unless otherwise mentioned, all the experimental conditions for podocyte cells were given for 48 h. The cell lysate was prepared for RNA isolation, immunoblotting, and enzyme-linked immunosorbent assay (ELISA). For immunofluorescence, cells were cultured on coverslips, followed by treatment as mentioned above, subsequent fixation with paraformaldehyde (4%), and blocking with PBS containing normal BSA (5%) before incubation with primary antibodies. The next day, the samples were incubated with Alexa Fluor-conjugated secondary antibodies, and DAPI for nuclear stain, for 1 h at room temperature. Images were acquired using a laser scanning microscope (ZEISS, Germany). For F-actin staining in podocytes cells essentially as described earlier^9^. Podocytes were incubated with fluorescent phalloidin–TRITC conjugate (P1951) for 40 min at room temperature. Next counterstaining by DAPI, mounting and images were acquired using a Leica trinocular microscope or Apotome Axio Imager Z2 (Zeiss). We analyzed images using LASX Industry Software and ImageJ (NIH). For live-cell imaging, cells were grown on µ-Dish 35 mm (#81156) at 60% confluency treatment performed for 48 h, and then imaged for 2 h on a Leica SP8 confocal laser scanning microscope with an HCX PL APO CS ×63, 1.40-NA oil immersion lens.

### Preparation of conditioned media and treatments

Differentiated human podocytes and HepG2 cells were treated with GH in the presence or absence of SB431542 and collected CM. CM was treated with an anti-GH antibody to neutralize residual GH activity. A fresh batch of differentiated podocytes was treated with 10, 25, or 50% of CM (percentage of CM prepared with the fresh RPMI media).

### Animal and tissues

Eight-week-old Swiss Webster male mice weighing 30 ± 5 g were used. Mice were allocated randomly (not blinded) into to five groups: (1) control group, (2) GH-treated group, (3) GH + DAPT-treated group, (4) GH + SB431542, and (5) GH + AG490. Mice received a single i.p. injection of hGH (1.5 mg/kg) or an equal saline volume per day for 6 weeks. We used a predefined value of *n* = 6 mice/group. The inhibitor groups were received DAPT (10 mg/kg body weight, b.w.), SB431542 (1 mg/kg b.w.), and AG490 (1 mg/kg of b.w.) per day in addition to GH. If animal dies prematurely the data excluded from the analysis. After 6 weeks of the experimental period, the mice were placed in individual metabolic cages for collecting 24 h urine to estimate albumin (#COD11573) and creatinine (#COD11502) levels, as recommended by the manufacturer’s protocol (Biosystems, Barcelona, Spain). Disease establishment was confirmed by estimating the UACR. An aliquot of urine from mice was subjected to SDS–PAGE gel, and silver staining was performed to compare the urinary protein profile for all five groups. Further, we have also measured the GFR in these mice, as described previously^9^. Mouse podocytes were isolated from the kidney of mice, as described in earlier protocol^48^. Briefly, glomeruli were prepared by filtration of the kidney’s cortex with mesh sieves, whose holes were 100, 76, and 54 μm in diameter. The tissues left on the mesh sieve with 54 μm holes were collected and prepared for the RT-PCR and immunoblotting. For histological analysis, the kidney cortex was fixed with 4% paraformaldehyde before embedding in paraffin. Paraffin-embedded tissues were sliced longitudinally into 3–4 μm thick sections, subjected to staining with PAS base, MT, and H&E staining. We obtained TEM images for glomerular sections from all the experimental mice groups described earlier^9^.

The generation of the homozygous GHR exon four floxed mice (Ghr^fl/fl^), pGHRKO mice, and heterozygous control (pGHR+/−) were generated, as described earlier^49^ in the laboratory of Prof. Ram Menon at the University of Michigan, Ann Arbor, MI. These mice were housed in a specific pathogen-free animal facility at the University of Michigan, and the University of Michigan IACUC approved all experiments pertinent to pGHR mice.

### Assessment of glomerulosclerosis

We performed MT staining to quantify glomerulosclerosis in 20 glomeruli (×400 magnification), and the average value from each mouse was presented as a data point. For the quantification of glomerulosclerosis, glomerular images were posterized using GNU Image Manipulation Program (GIMP) to isolate the blue color, which indicates fibrosis. Next glomerular fibrotic area was quantified using ImageJ (NIH) in a double-blinded fashion. We performed PAS staining to quantify glomerular aberrations, such as mesangial matrix, glomerular capillaries, and glomerular tuft area. We analyzed 20 glomeruli from each mouse (×400 magnification) using ImageJ in a double-blinded fashion, and the average value from each mouse was presented as a data point in the plot.

### RNA extraction and quantitative RT-PCR assay

The total RNA was isolated from human podocytes and mouse podocytes using a TRIzol reagent. Next, 1 μg of total RNA was reverse transcribed using the cDNA synthesis kit (#6110 A, PrimeScript 1st strand cDNA Synthesis kit, TaKaRa). The qRT-PCR analysis was performed by the QuantStudio 3 system (Applied Biosystems) with SYBR Green (Kappa Biosystem) Master Mix as mentioned in the following protocol: initial denaturation at 95 °C for 3 min, followed by 35 cycles of three steps each at 95 °C for the 30 s, 60 °C for 30 s, and 72 °C for 30 s. The expression levels of β-actin did not change under the different experimental conditions and were used as an internal control to normalize each gene’s mRNA expression. The primers used are shown in Supplementary Table.1.

### Immunoblotting

Cytoplasmic extract for immunoblotting was prepared as described previously^9^. Briefly, human podocytes and isolated mouse primary podocytes were lysed by RIPA buffer (#9806, Cell Signaling Technology) containing protease inhibitor mixture (#05056489001, Sigma Aldrich) and phosphatase inhibitor tablets (Roche), centrifuged and collected as supernatants. However, for nuclear extract protein sample preparation, pellet was resuspended with 20 mM HEPES (pH 7.9), 25% glycerol, 0.42 M NaCl, 0.2 mM EDTA, 1.5 mM MgCl2, 1 mM DTT, and 0.2 mM PMSF) and vortexed for 20 s. Incubate the cell lysate for 25 min on ice and vortex every 10 min for 10 s. Next, cell lysate was centrifuged for 12 min at 19,400 × *g* at 4 °C, and the supernatant (nuclear) was collected. The protein concentrations of cell and mouse podocyte lysates were determined using a bicinchoninic acid reagent (Thermo Scientific), using BSA as a standard. An equal amount of protein from corresponding samples was subjected (20–25 μg) to SDS–PAGE and immunoblotting, and bands were visualized using a ChemiDocTM XRS System (Bio-Rad, Hercules, CA).

### Enzyme-linked immunosorbent assay

γ-Secretase activity was assessed as described earlier^9^. Briefly, human podocytes were treated with or without GH, TGF-β1, GH + DAPT, TGF-β1 + SB431542, GH + SB431542, and GH + AG490 for 48 h. After the treatment period, the cleavage-dependent release of APH-1A measured at 450 nm using a fluorescent microplate reader (Multiskan O Microplate reader, ThermoFisher Scientific). We estimated TGF-β1 levels in human samples using TGF-β1 ELISA kit (R&D Systems), and mice samples using Mouse TGF-β1 ELISA Kit (ab119557).

### Cellular DNA flow cytometric analysis

The single-cell suspension of human podocytes (5 × 10^5^–1 × 10^6^ cells) from with or without GH, TGFβ1, GH + DAPT, TGF-β1 + SB431542, GH + SB431542, and GH + AG490 for 48 h were prepared in 300 μl PBS, fixed by cold 70% ethanol for 30 min at 4 °C, and then washed and resuspended in 300 μl PBS, followed by treatment with 3 μl RNase at 37 °C for 30 min, chilled on ice, and 30 μl propidium iodide (PI; Roche) treatment in the dark at room temperature for 1 h. DNA contents were acquired by a S3e Cell Sorter flow cytometer (Bio-Rad) using FCS Express 7 program.

### Apoptosis analysis in podocytes

Apoptotic cell death was measured by an Alexa Fluor® 488 Annexin V and PI apoptosis detection kit (ThermoFisher Scientific), according to the manufacturer’s protocol. Briefly, human podocytes were plated on 6 cm dishes at 1 × 10^5^ cells per dish with or without GH, TGF-β1, GH + DAPT, TGF-β1 + SB431542, GH + SB431542, and GH + AG490 for 48 h. Next podocytes were harvested with the help of trypsin, washed with cold PBS twice, resuspended in binding buffer, and stained with FITC-Annexin V and PI in the dark at room temperature for 15 min. After incubation, a binding buffer was added, and the podocytes were analyzed by a S3e Cell Sorter flow cytometer (Bio-Rad). Unstained cells and cells stained with FITC-Annexin V or PI alone were used as controls to set up compensation and quadrants in flow cytometry. The FCS Express 7 program analyzed the results.

### Reporter assays

HES1 promoter activity luciferase assay was performed, as described earlier^50^. Briefly, podocytes were transfected with a pHES1(467)-Luc (procured from Addgene) and internal control expressing the Renilla luciferase, pRL‐TK (Promega). Podocytes were transfected using Xfect polymer (DSS Takara Bio, New Delhi, India), as per the manufacturer’s instructions. After 48 h of transfection, cells were treated with or without GH, TGF-β1, GH + DAPT, TGF-β1 + SB431542, GH + SB431542, and GH + AG490 for 48 h, and washed twice with PBS and harvested in 100 μl of passive lysis buffer. After a brief freeze‐thaw cycle, the insoluble debris was removed by centrifugation at 12,000 × *g* (4 °C for 5 min), and 20 μl of supernatant was used for luciferase assay. The activity of the co-transfected luciferase reporter plasmid was used to normalize transfection efficiency.

SMAD4 Cignal-GFP reporter assay was performed in podocyte cells, according to the earlier published protocols with minor modifications^51^. Briefly, podocyte cells in a 96-well plate, 1000 cells/well were seeded before the day of transfection. Cells were transfected either with SMAD4-GFP, positive control, and negative control vectors in triplicates. After transfection cells were left untreated in a complete medium for 16 h. Then cells were treated with GH-CM, anti-TGFβ1 antibody (#AB-100-NA), GH, and TGFβ1. After 48 h of treatment, we took images using a Leica trinocular microscope. The fluorescence emission spectrum for these samples was acquired at 510–520 nm by using a fluorescent microplate reader (Multiskan O Microplate Spectrophotometer, ThermoFisher Scientific).

For SMAD4 luciferase assay, podocyte cells were seeded into a six-well plate (2 × 10^5^ cells per well). The cells were then co-transfected with either 0.3 µg/well of Smad4 firefly luciferase reporter plasmid constructs (pLuc366 or pLuc207) or the control pGL3-Basic vector (Promega, San Luis Obispo, USA). The renilla luciferase plasmid was also co-transfected to correct variations in transfection efficiency (45 ng/well). After incubating for 24 h, we gave treatment for 48 h. Next cells were harvested from each experimental condition, and the luciferase activity was measured using a fluorescent microplate reader (Multiskan O Microplate reader, ThermoFisher Scientific). The final activity was calculated as the ratio of firefly luciferase activity versus renilla luciferase activity units.

### Transfection of podocytes for knockdown and overexpression

Human podocytes were transfected with siRNA, as described earlier^50^. Briefly, transfection was done using jetPEI reagent (Polyplus, Illkirch, France). Podocytes were seeded at 70–90% confluency in six-well plates and transiently transfected with siRNA specific to TGFBR1 (#EHU051131). After 72 h of transfection, cells were treated with or without GH, TGF-β1, GH + DAPT, TGF-β1 + SB431542, GH + SB431542, and GH + AG490 for 48 h. Next, cells were washed twice with PBS and lysed with RIPA buffer; the expression levels measured by immunoblotting as described above. In addition, podocytes were transfected with an overexpressing vector from Addgene (#86500; pT3-EF1aH NICD1) and its empty parental vector (pT3-EF1aH) using Xfect polymer (DSS Takara Bio, New Delhi, India), as per the manufacturer’s instructions. After 24 h of transfection incubation, cells were harvested, and immunoblotting was performed. Similarly, we transfected podocytes with siRNA for GHR (#EHU060521) with appropriate controls.

### Immunohistochemistry of human kidney biopsies

Kidney biopsies were not performed for the purpose of this study, but collected from archived kidney biopsies without patients identifiers from Guntur Medical College, AP, India. Informed consent was obtained from the patients that these kidney biopsies obtained for patient care could be used for research purpose. The selected cases were with biopsy proven DN and proteinuria.

### Statistical analysis

Statistical analysis methods are detailed in the figure legends. There were no studies in which investigations were blined. The sample size for each experiment was chosen based on published literature with a similar methodology^9^. Two-group comparisions were performed with Student’s *t* test. Multiple groups comparision were examined for statistical significance using one-way ANOVA. GraphPad Prism 6 (GraphPad, San Diego, CA) was used for the data analysis.

## Supplementary information

Supplementary data

Live cell imaging of control podocytes

Live cell imaging of GH-treated podocytes

Apoptosis of GH-treated podocytes
