# Supplementary material for: Growth hormone induces mitotic catastrophe of glomerular podocytes and contributes to proteinuria
Source: Cell Death Dis. 2021 Apr 1;12(4):342. doi: 10.1038/s41419-021-03643-6 (PMC8016968; doi:10.1038/s41419-021-03643-6)

Supplementary Data

Growth hormone induces mitotic catastrophe of glomerular podocytes and contributes to proteinuria

Rajkishor Nishad[#1], Dhanunjay Mukhi[#1], Ashish Kumar Singh[1], Manga Motrapu[1], Kumaraswami Chintala[1], Prasad Tammineni[2], Anil K Pasupulati[1]

[1]Department of Biochemistry & [2]Animal Biology, School of Life Sciences, University of Hyderabad, Hyderabad, India-500046.

# Equal contribution

Supplementary Figure Legends:

Supplementary Figure 1: Demonstration of podocyte differentiation and confirmation of GHR expression in podocytes: (a) Light microscope images. Magnification x200. Scale bar=50μm. Podocytes proliferate at growth permissive temperature (33oC) and differentiated for 14 days at non-permissive temperature (37oC). Undifferentiated (left panels) and differentiated (right panels) podocytes. **(b)** Phalloidin staining showing the complete development of actin stress fibers in differentiated podocytes. Magnification x630. Scale bar=20μm. **(c)** Immunofluorescence and **(d)** immunoblotting for nephrin and podocin expression in undifferentiated podocytes vs differentiated podocytes. The white arrow indicates the specific expression of nephrin and podocin. Magnification x630. Scale bar=20μm. Growth hormone receptor (GHR) expression in **(e)** HepG2 cells, **(f)** podocytes, **(g)** mouse glomerulus, and **(h)** human glomerulus. GHR (green colour), DAPI (blue colour), and Nephrin (red colour). The white asterisk indicates the specific expression of GHR. Magnification x630. Scale bar=20μm.

Supplementary Figure 2: GH induces TGF-β1 in both HepG2 cells and podocytes: (a) qRT-PCR analysis showing the expression of TGF-β1 in HepG2 cells treated with GH (500 ng/ml) for indicated time points. Expression levels were normalized to β-Actin mRNA levels and presented as fold-change. Mean±SD, (n=5). ****p<0.0001 by 1-way ANOVA post hoc Dunnett test. **(b)** Immunoblotting analysis for indicated proteins from HepG2 cells treated with GH in concentration (500 ng/ml) for 48h (n=3). **(c)** Heat map presenting the differential expression of 14 genes in GH treated podocytes. Each vertical axis represents the gene rank with log2 fold change values. The colour intensity of each row represents a mean-centred log2 expression. We used the Gene pattern (http://genepattern.broadinstitute.org/gp/) to generate this heat map. The original microarray data is available at the GEO repository (GSE21327). **(d&e)** Immunoblotting analysis for indicated proteins from HepG2 and podocytes treated with 10 to 50% conditioned media (GH-CM), GH-CM+SB (SB indicates SB431542, an inhibitor for TGFBR1) and GH-CM-Ab (Ab indicates TGF-β1 neutralizing antibody) for 48h. (n=3).

Supplementary Figure 3: GH induced TGF-β1 elicit signalling in both autocrine and paracrine manner: (a) Immunofluorescence for the nuclear localization of SMAD4 in podocytes treated with GH, TGF-β1, and conditioned medium (CM). Con-CM; CM from cells naive to GH. GH-CM; CM from GH (500 ng/ml) treated podocytes. GH-CM+SB; CM from GH (500 ng/ml) treated podocytes supplemented with SB (TGFBR1 inhibitor). Magnification x400. Scale bar=50 μm. (n=3).

Ab; **(b)** SMAD4 luciferase activity in podocytes treated with (48h) or without a conditioned medium from indicated experimental conditions. GH-CM+Ab; CM from GH treated podocytes neutralized with an anti-GH antibody. Mean±SD. (n=6). ****p<0.0001 by Student's t-test. **(c)** SMAD-GFP reporter assay was performed in HEK293T cells treated with GH, CM-GH, and rTGF-β1. Un-transfected indicates there is no vector backbone. Negative control indicates an empty vector backbone (Cignal SMAD4 without GFP). Positive control indicates Cignal SMAD4-GFP under treatment with 5 ng/ml TGF-β1. **(d)** Immunoblot for indicated proteins in podocytes exposed to CM-GH for 48h. β-Actin served as an internal loading control for western blots. (n=3). **(e)** γ-secretase activity was measured in podocytes treated with or without GH (500ng/ml), TGF-β1 (5ng/ml), GH+DAPT (5μg/ml), TGF-β1 +SB431542 (100nM/ml), TGF-β1+DAPT, GH+SB431542 and GH+AG490 (10μM/ml). Mean±SD. (n=6). ****p<0.0001 by Student's t-test. 50% of CM was used to treat the cells in all the above experiments.

Supplementary Figure 4: GH induces podocyte cell cycle re-entry and apoptosis. (A) Immunoblotting analysis for CyclinB1 in human podocytes treated with GH in concentration (500 ng/ml) and time (up to 72h). (B) Live cell images representing the GH treatment induces podocyte cytokinesis failure. Yellow arrowhead indicates the site of initial cytokinetic ring formation and later incomplete cytokinesis. (C) Live-cell images of podocytes treated with or without GH treatment. White arrowhead indicates the podocyte naïve to GH treatment, whereas red arrowhead indicates that the podocyte is exposed to GH and undergoing apoptosis.

Supplementary Figure 5: Amelioration of GHR protects from GH-induced mitotic catastrophe in vitro and proteinuria in vitro: (A&B) qRT-PCR analysis showing the expression of Bax and Bcl2 in human podocytes exposed to various treatment conditions as indicated for 48h. mRNA levels were normalized to β-Actin and presented as fold-change on the y-axis. Mean±SD. (n=5). ****p<0.0001 by Student's t-test. **(C)** Immunofluorescence staining for Caspase 3 (red colour) and counterstained with DAPI (blue colour) in podocytes exposed to various conditions indicated for 48h. Magnification x630. Scale bar=20 μm. (n=3). **(d)** Podocytes were transfected with GHR-siRNA and scrambled RNA and treated with or without GH, and analyzed by immunoblotting for indicated protein markers, (n=3). **(e)** Mice with conditional knock-out of podocyte GHR (pGHR-/-) were generated. Urinary albumin creatinine ratio (UACR) was estimated in pGHR+/- and streptozotocin (STZ) injected pGHR+/- and pGHR-/- mice. Mean±SD. ****p<0.0001 by Student's t-test. Each data point represents the average value of three independent experiments from a single mouse. **(f)** Silver staining was performed on the urine samples from pGHR+/- and STZ injected pGHR+/- and pGHR-/-

mice. BSA; Bovine Serum Albumin, M; protein standard marker. **(g)** NICD1 expression in glomerular sections from pGHR+/- and STZ injected pGHR+/- and pGHR-/- mice. White arrowhead indicating the specific expression of NICD1. NICD1 (red colour) and DAPI (blue colour). Magnification x630, Scale bar=20μm.

Supplementary Figure 6: Blockade of GHR and TGFBR1 prevent podocyte injury and glomerulosclerosis. (a) Immunoblots for indicated proteins in mouse primary podocytes (MPC) from control and treatment groups. (n=3). **(b)** Quantification of TGF-β1 in urine from control and treatment groups. Mean±SD. (n=6) ****$p<0.0001$ by Student t-test. Each data point represents the average value of a single mice from each group (n=6). (**c**) The extent of glomerulosclerosis as Masson's trichrome stained positive area in indicated experimental conditions.

Supplementary Table 1: The list of qRT-PCR primers used in this study

TGFBR1(Human)	FP= TCAATTGTAAGCACATTGAAAGGG
	RP=TTCGCCCGGCAGATCTAAAC
TGFBR1(Mouse)	FP=AAGACAACTGCCAGCCCTTAG
	RP=TCATTTAGTGCCACACCCCA
TGF-β1(Human)	FP=GTTCAGGTACCGCTTCTCGG
	RP=CCTGATCGCCTCCCTTCATTT
TGF-β1(Mouse)	FP=AAATCAACGGGATCAGCCCC
	RP=CGCACACAGCAGTTCTTCTC
Notch1(Human)	FP=TGAATGGCGGGAAGTGTGAA
	RP=CACAGCTGCAGGCATAGTCT
Notch1(Mouse)	FP=AGACATGTAGGGCAGTCAGC
	RP=CCAGAGCTTACGTCATCCCA
HES1(Human)	FP=ATGACAGTGAAGCACCTCCG
	RP=GAGTGCGCACCTCGGTATTA
HES1(Mouse)	FP=TCCCACGGTCTGGGTCTTAT
	RP= GTGCTAAACCACTGACCCCT
JAG1(Human)	FP=CCTGTCCATGCAGAACGTGA
	RP=CGCGGGACTGATACTCCTT
JAG1(Mouse)	FP=GTTTCGCAGGAGGCCTGTTT
	RP=CTGGGTCAGCACCGAGAATG
BAX(Human)	FP=CTGACGGCAACTTCAACTGG
	RP=GCAGGGGGTTGATACCACG
Bcl2(Human)	FP=CGGGTTGTCGCCCTTTTCTA
	RP=TCACAGATCTGAGGGGGAGC
CTGF(Mouse)	FP=GCATCTCCACCCGAGTTACC
	RP=TAGGGGCAGAGGATGTACCTT
BMP7(Mouse)	FP=GTCTGCCAGGAAAGTGTCCA
	RP=CGAGGCTTGCGATTACTCCT

Supplymentary Fig 1: Demonstration of podocyte differentation and confirmation of GHR expression in podocyte.

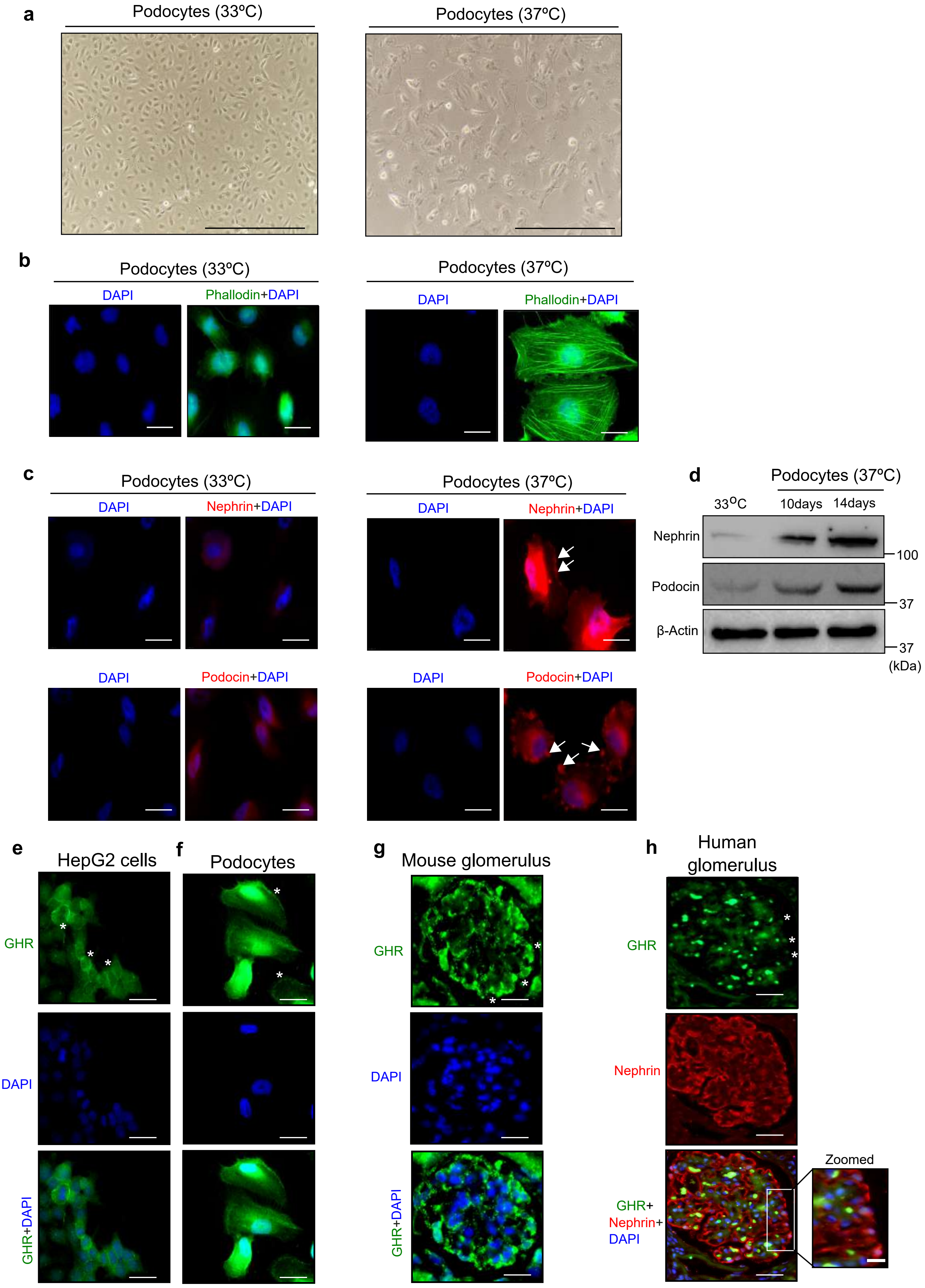

Supplementary Figure 2: GH induces TGF-β1 in both HepG2 cells and podocytes.

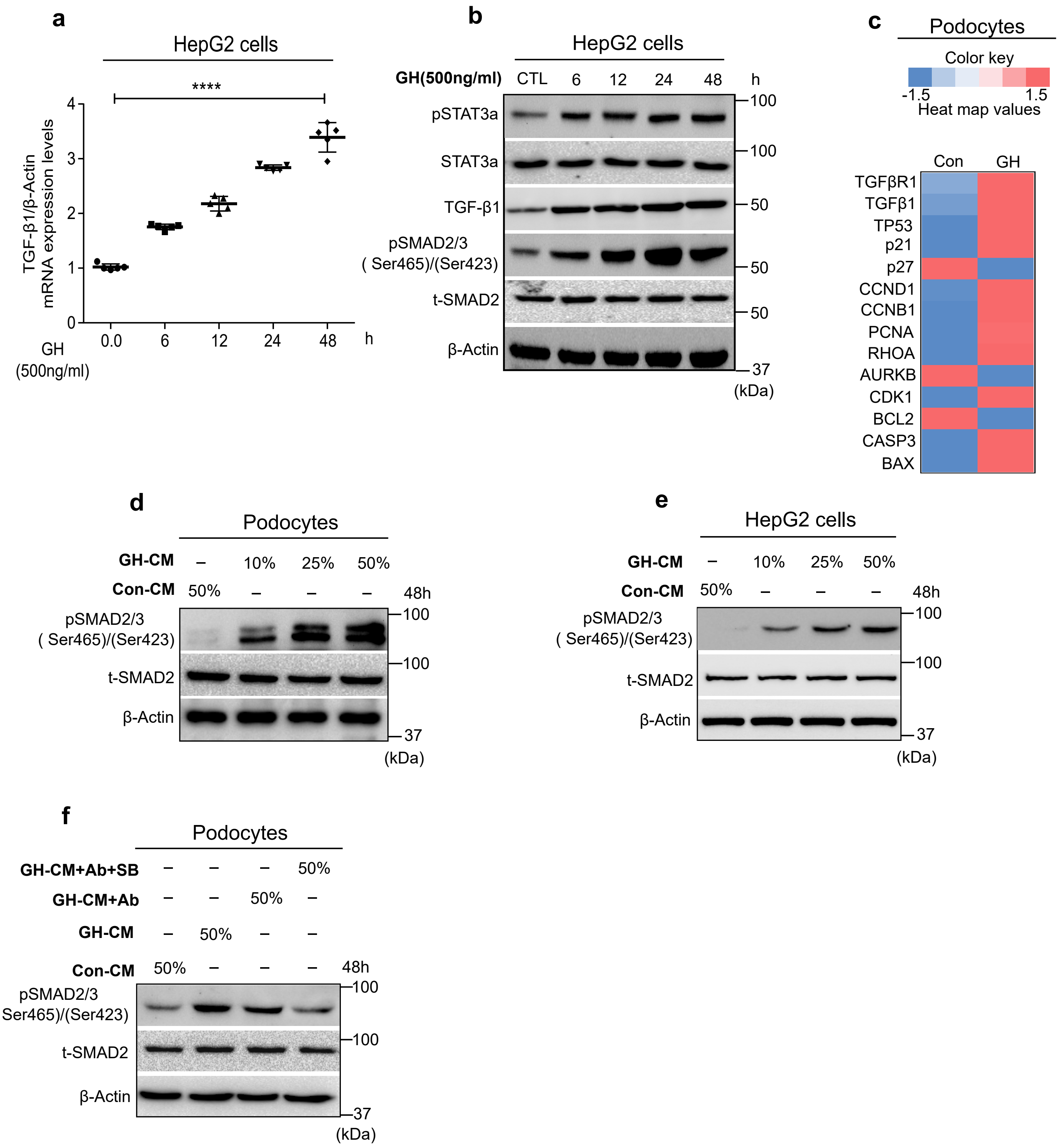

Supplementary Fig 3: GH induced TGF-β1 elicit signaling in both autocrine and paracrine manner.

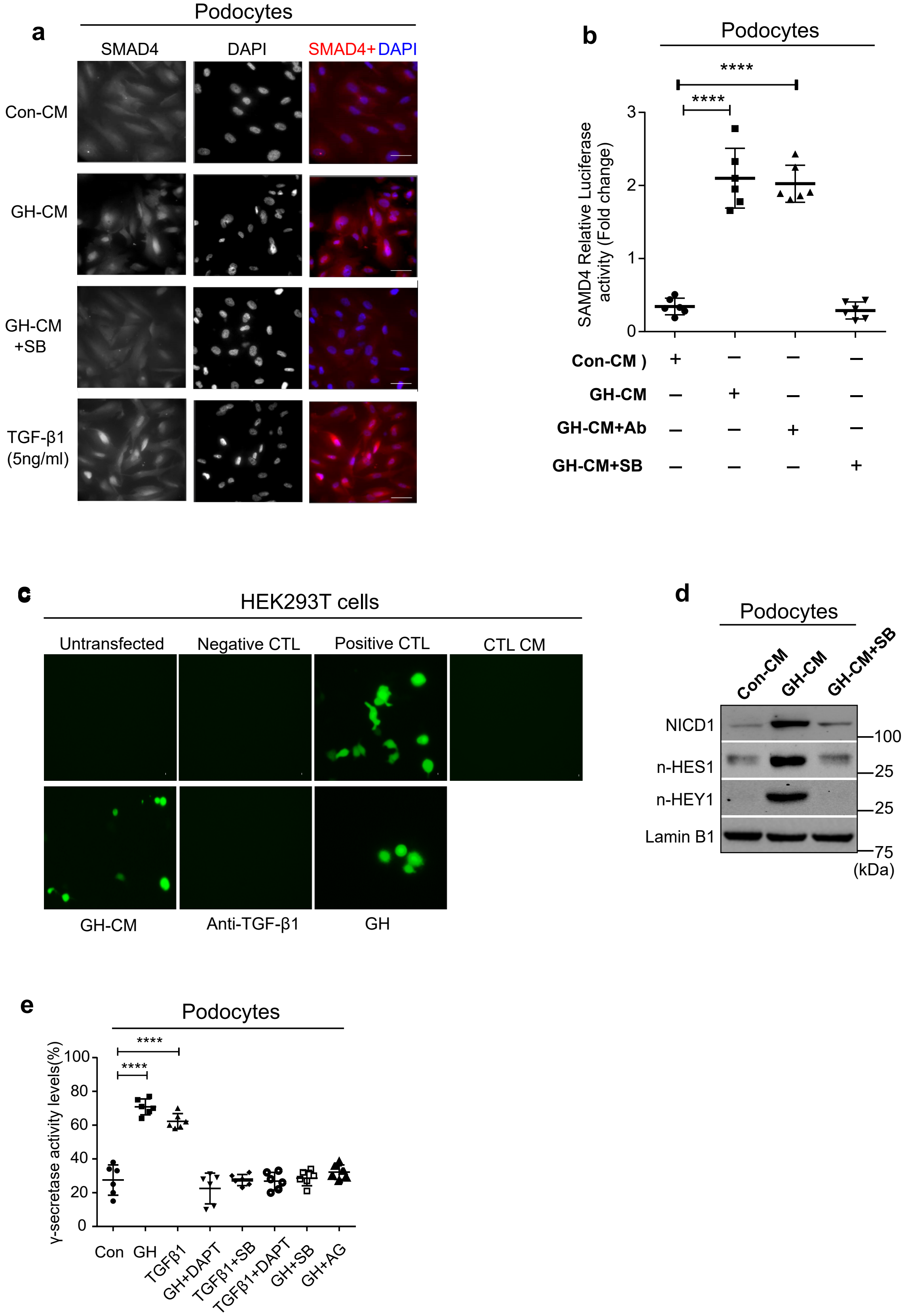

Supplementary Fig 4: GH induces podocyte cell cycle re-entry and apoptosis.

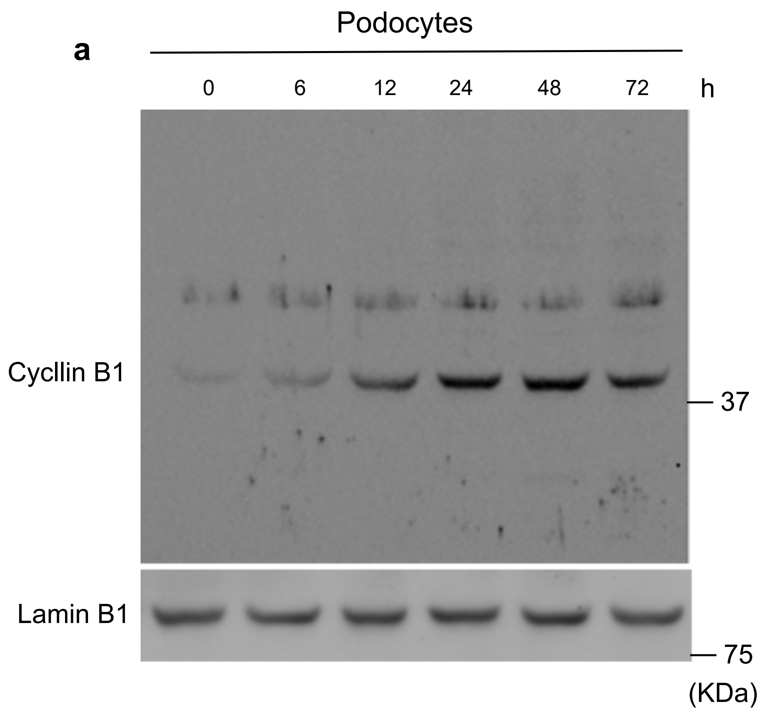

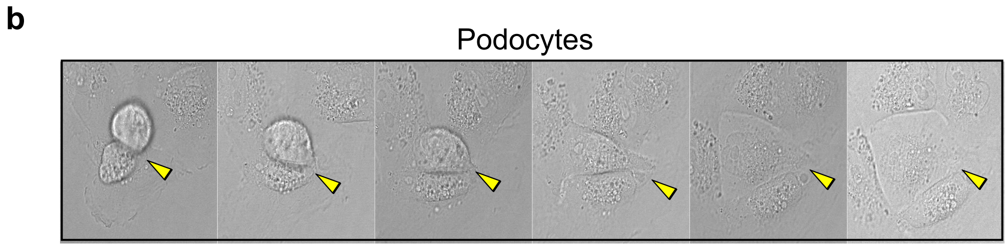

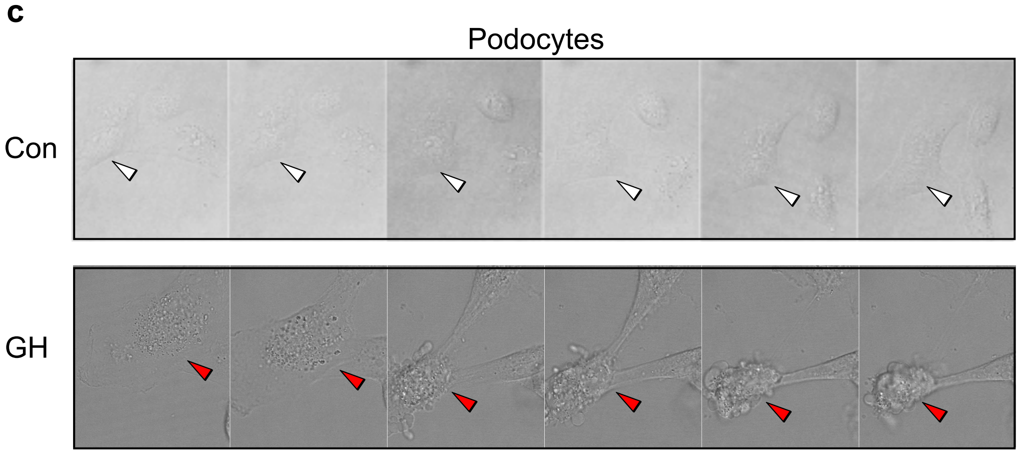

Supplementary Fig 5: Amelioration of GHR protects from GH-induced mitotic catastrophe *in vitro* and proteinuria *in vivo.*

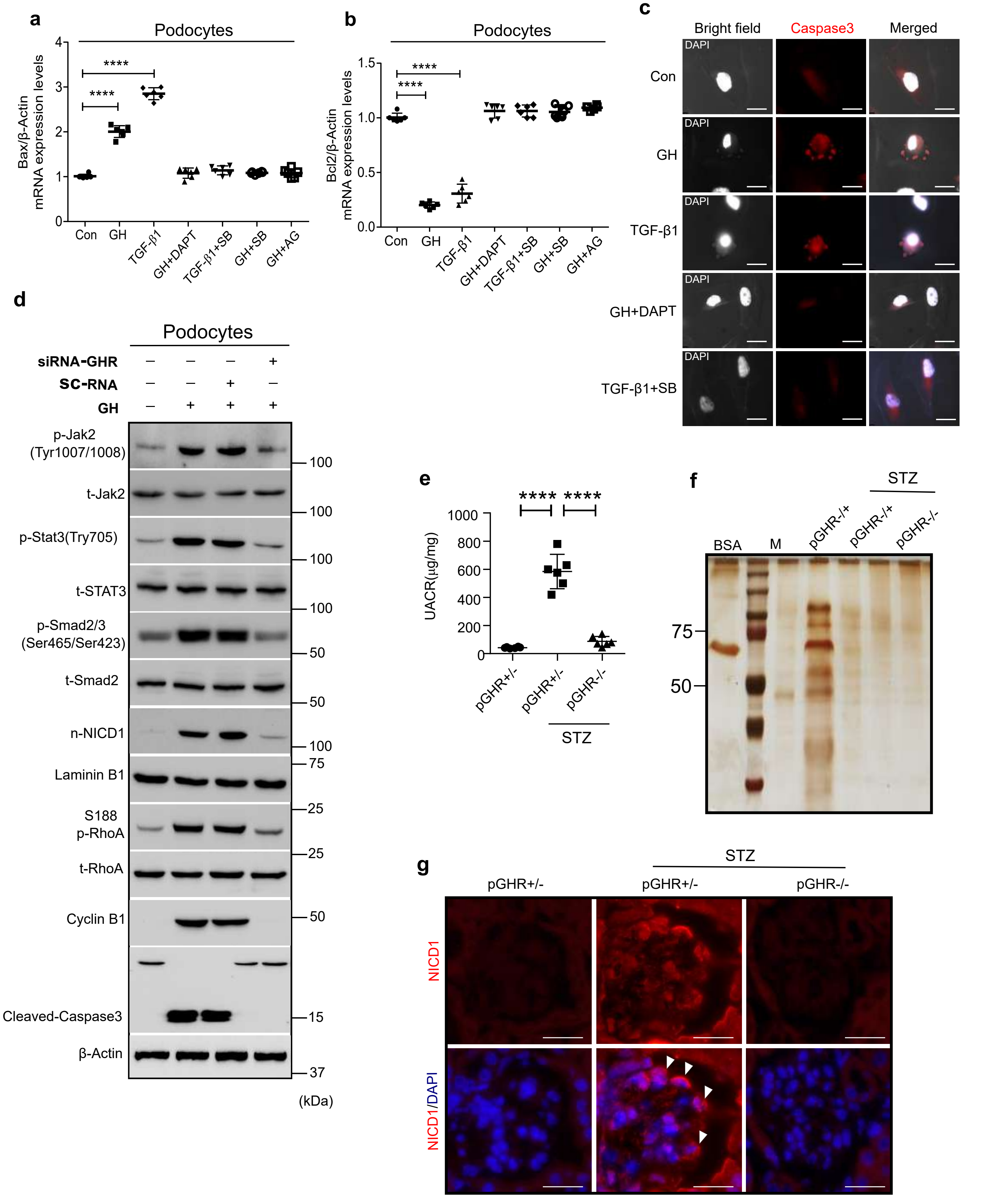

Supplementary Fig 6: Blockade of GHR and TGFBR1 prevent podocyte injury and glomerulosclerosis.

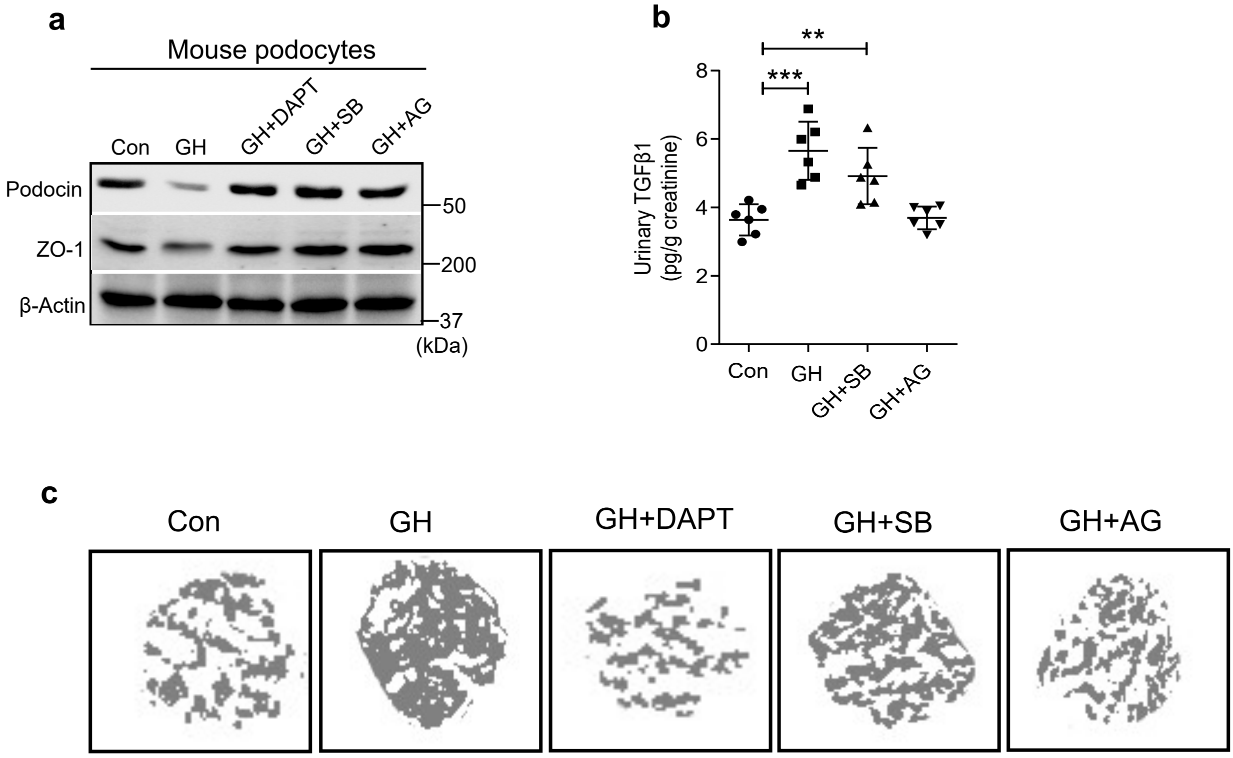

Supplement: Supplementary file 1 — Supplementary data [file 41419_2021_3643_MOESM1_ESM.pdf]
